# Supplementary figures and images for: Hyperacetylated histone H4 is a source of carbon contributing to lipid synthesis
Source: EMBO J. 2024 Feb 21;43(7):4. doi: 10.1038/s44318-024-00053-0 (PMC10987603; doi:10.1038/s44318-024-00053-0)

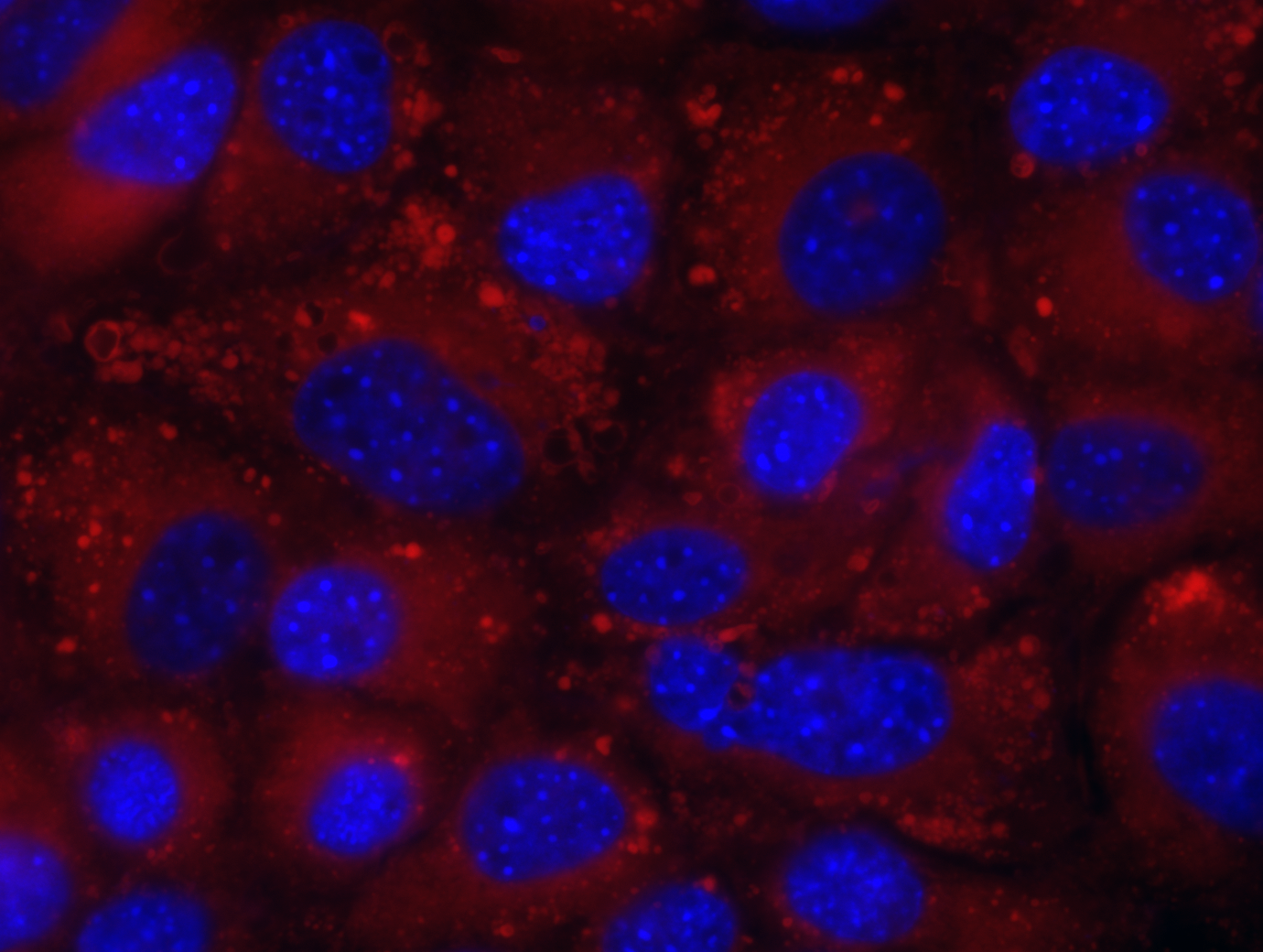

Supplement: Supplementary file 1 — Source Data Fig. 1 [file 44318_2024_53_MOESM1_ESM.zip › SD Figure 1/1D/Nile Red_Images/GCN5-KD.tif]

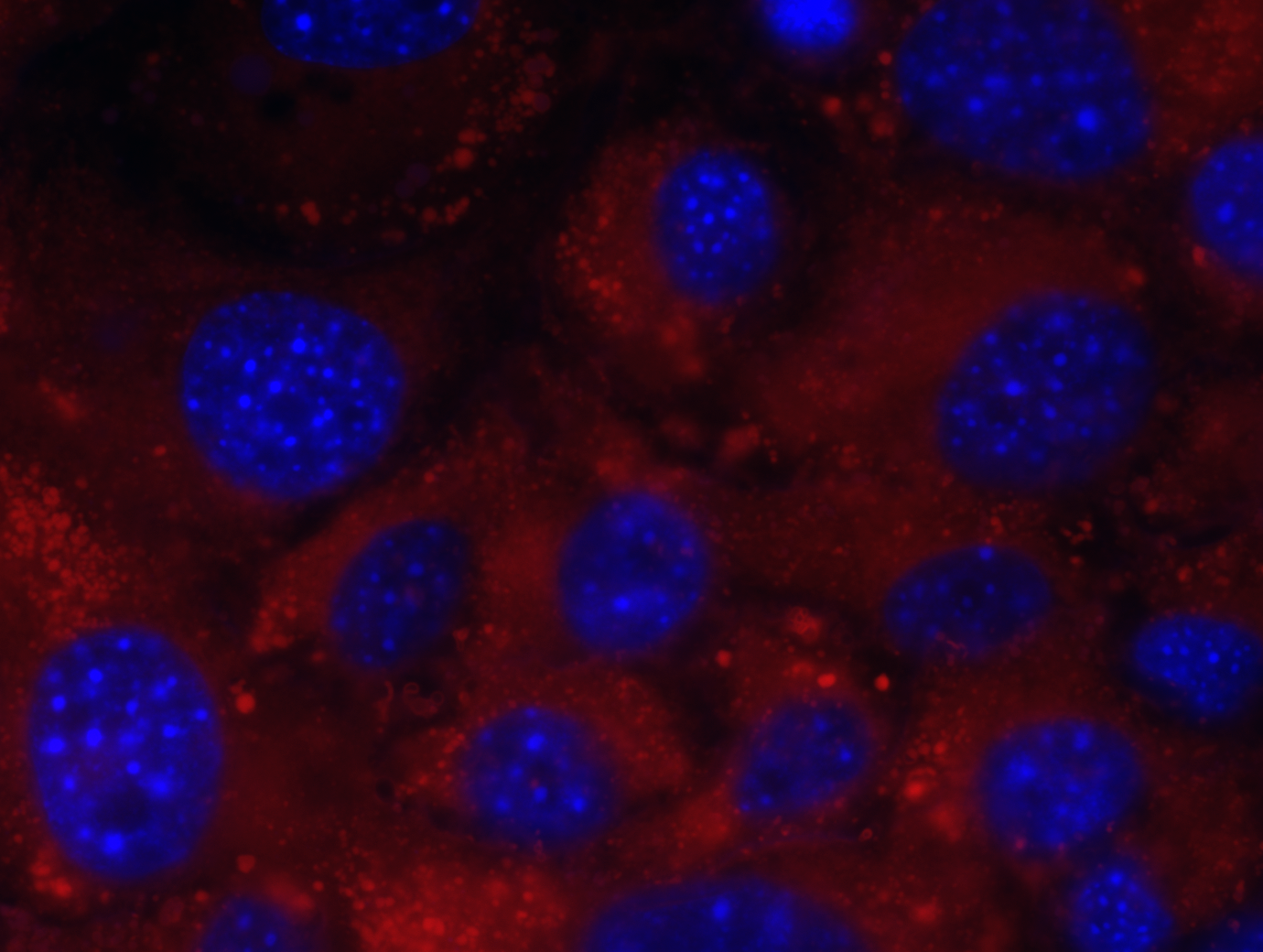

Supplement: Supplementary file 1 — Source Data Fig. 1 [file 44318_2024_53_MOESM1_ESM.zip › SD Figure 1/1D/Nile Red_Images/MYST1-KD.tif]

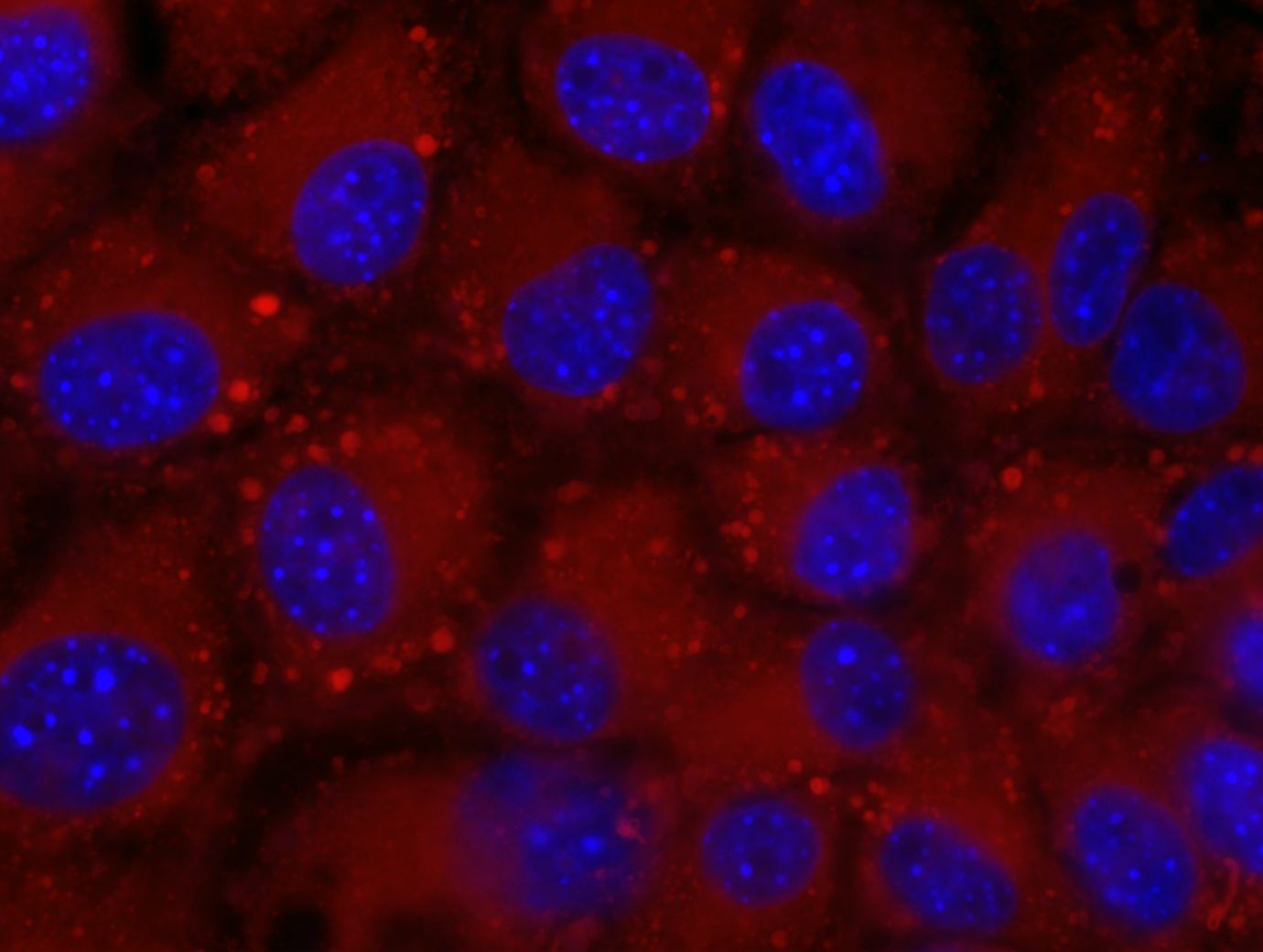

Supplement: Supplementary file 1 — Source Data Fig. 1 [file 44318_2024_53_MOESM1_ESM.zip › SD Figure 1/1D/Nile Red_Images/NAA10-KD.tif]

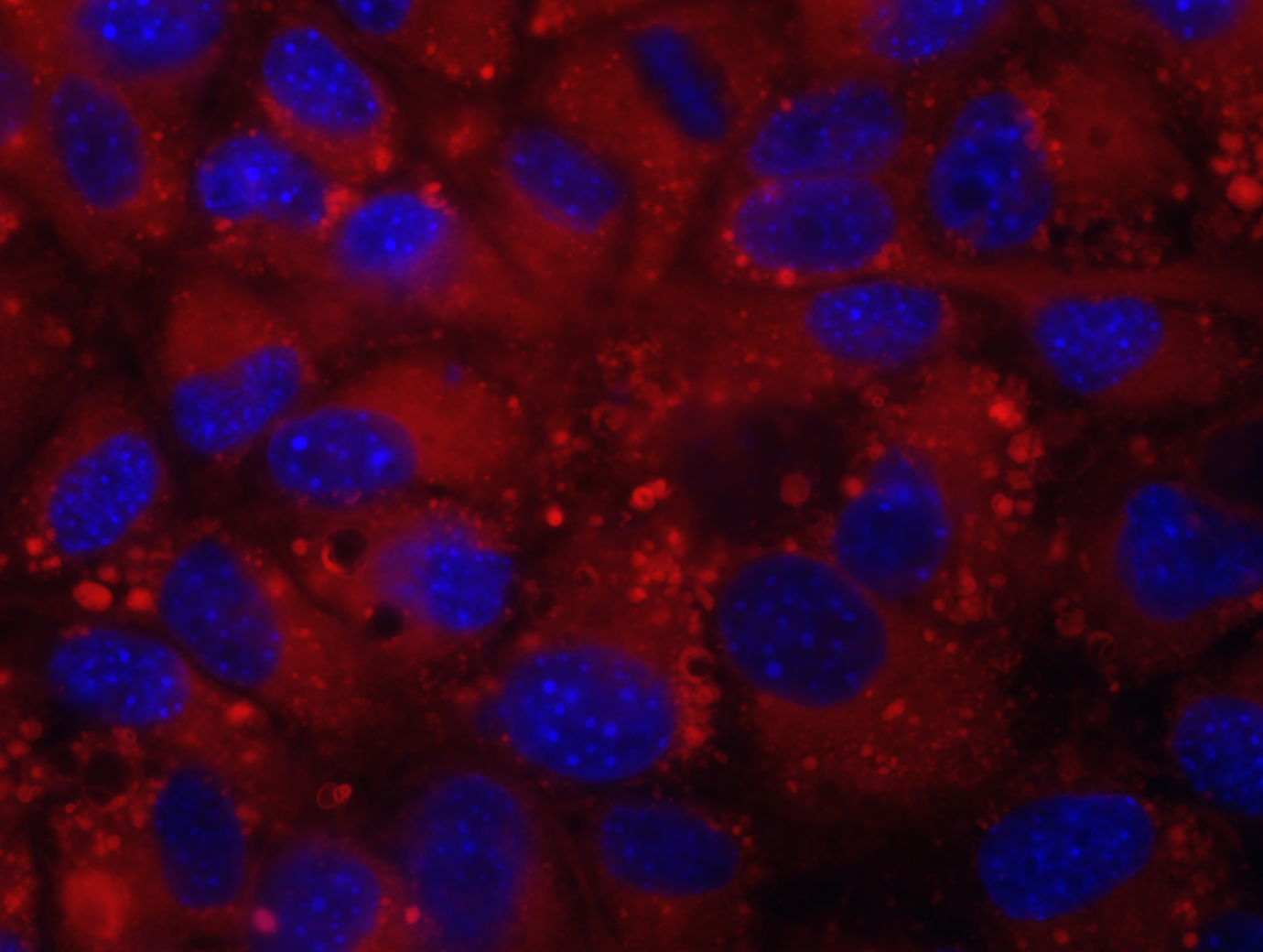

Supplement: Supplementary file 1 — Source Data Fig. 1 [file 44318_2024_53_MOESM1_ESM.zip › SD Figure 1/1D/Nile Red_Images/NAA40-KD.tif]

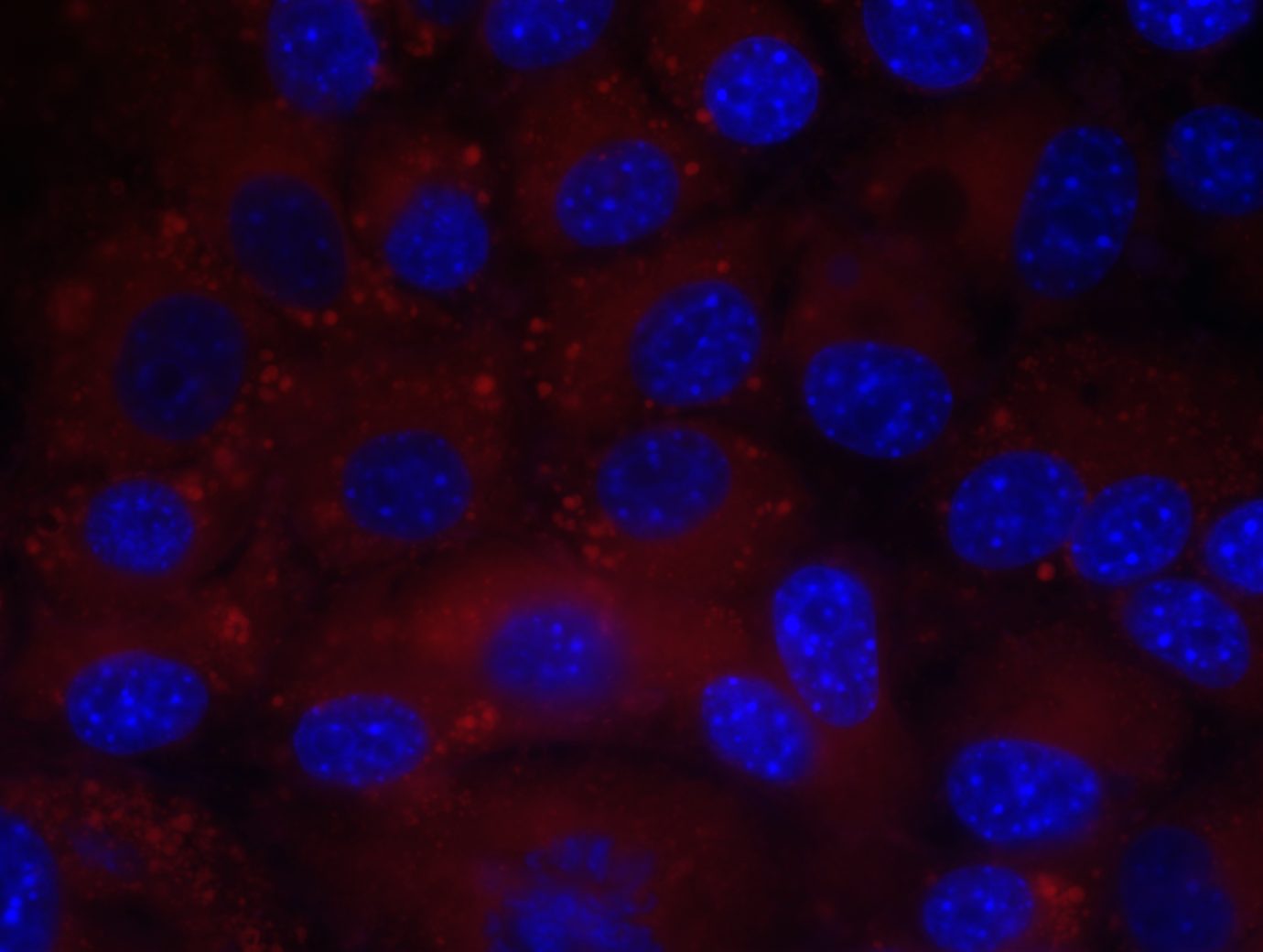

Supplement: Supplementary file 1 — Source Data Fig. 1 [file 44318_2024_53_MOESM1_ESM.zip › SD Figure 1/1D/Nile Red_Images/Scramble.tif]

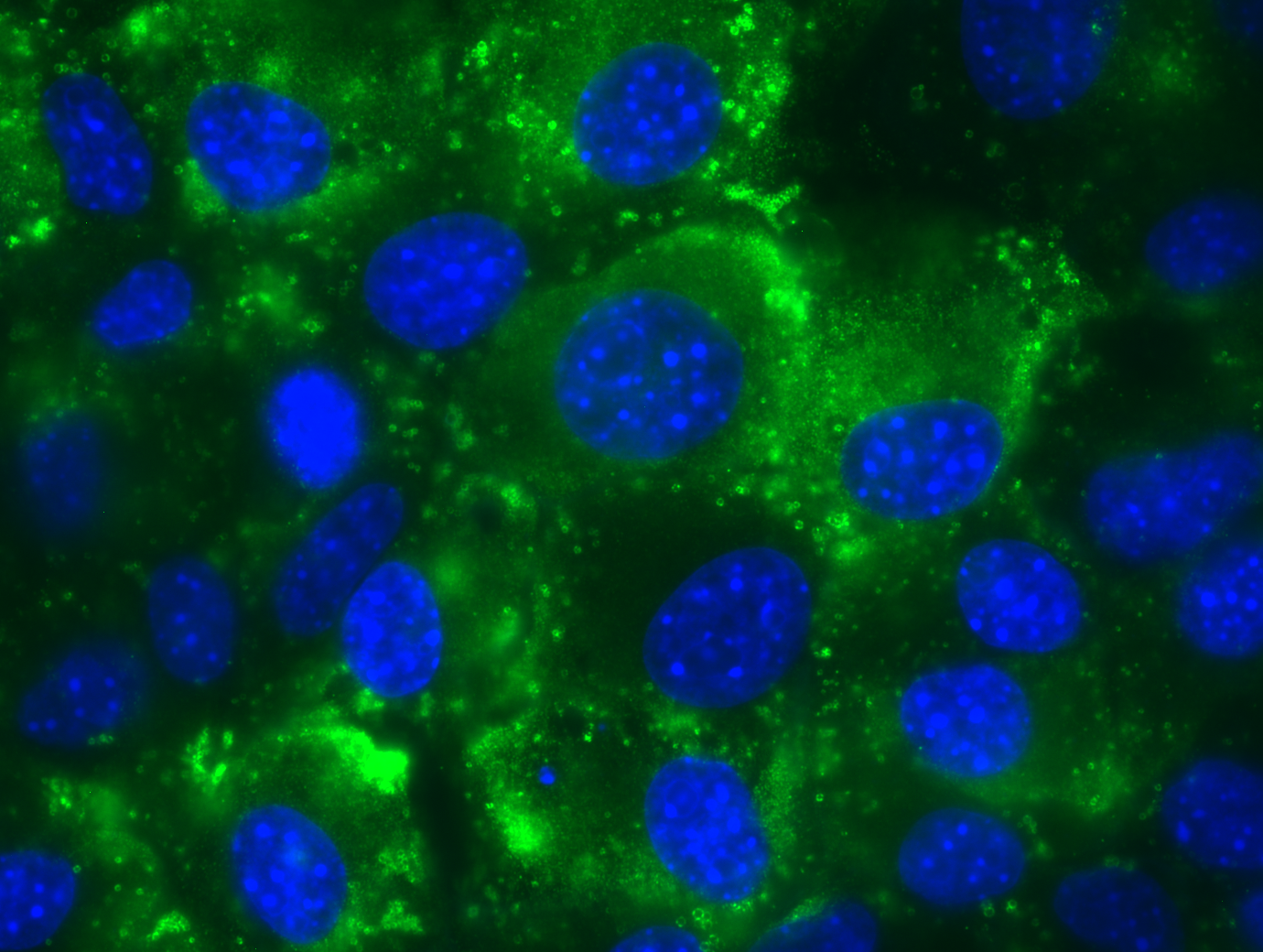

Supplement: Supplementary file 1 — Source Data Fig. 1 [file 44318_2024_53_MOESM1_ESM.zip › SD Figure 1/1D/Perilipin-1_Images/GCN5-KD.TIF]

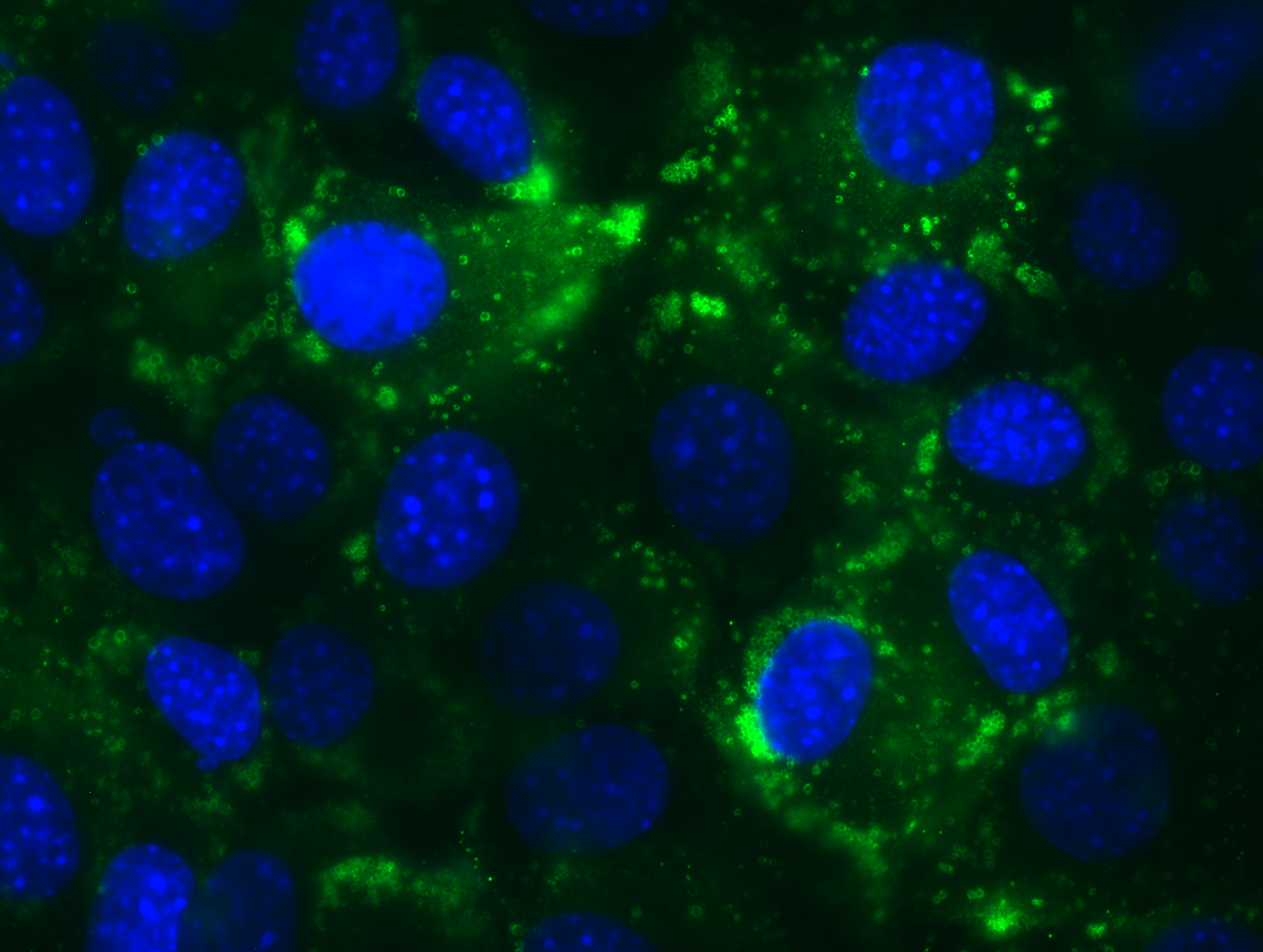

Supplement: Supplementary file 1 — Source Data Fig. 1 [file 44318_2024_53_MOESM1_ESM.zip › SD Figure 1/1D/Perilipin-1_Images/MYST1-KD.TIF]

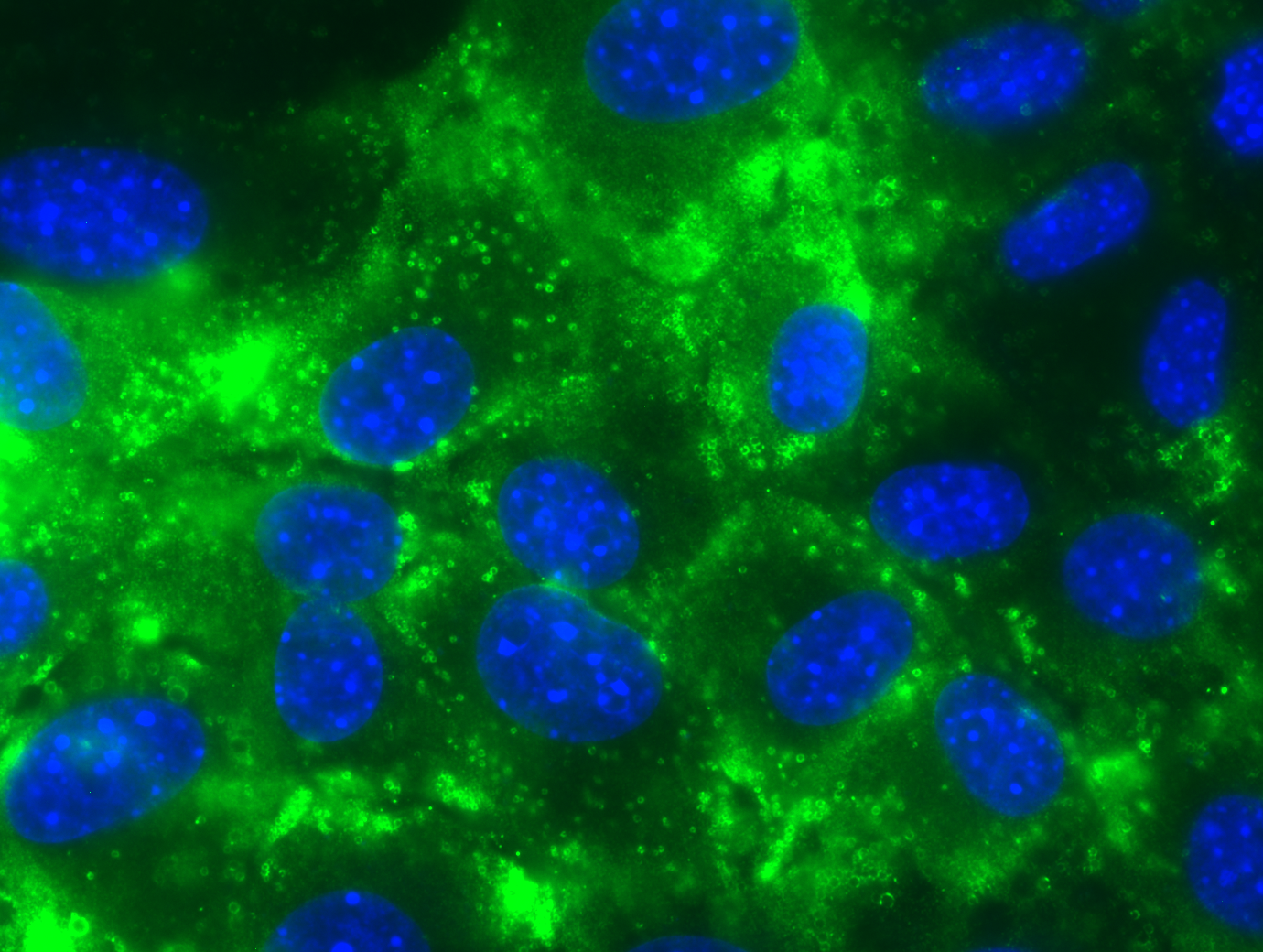

Supplement: Supplementary file 1 — Source Data Fig. 1 [file 44318_2024_53_MOESM1_ESM.zip › SD Figure 1/1D/Perilipin-1_Images/NAA10-KD.TIF]

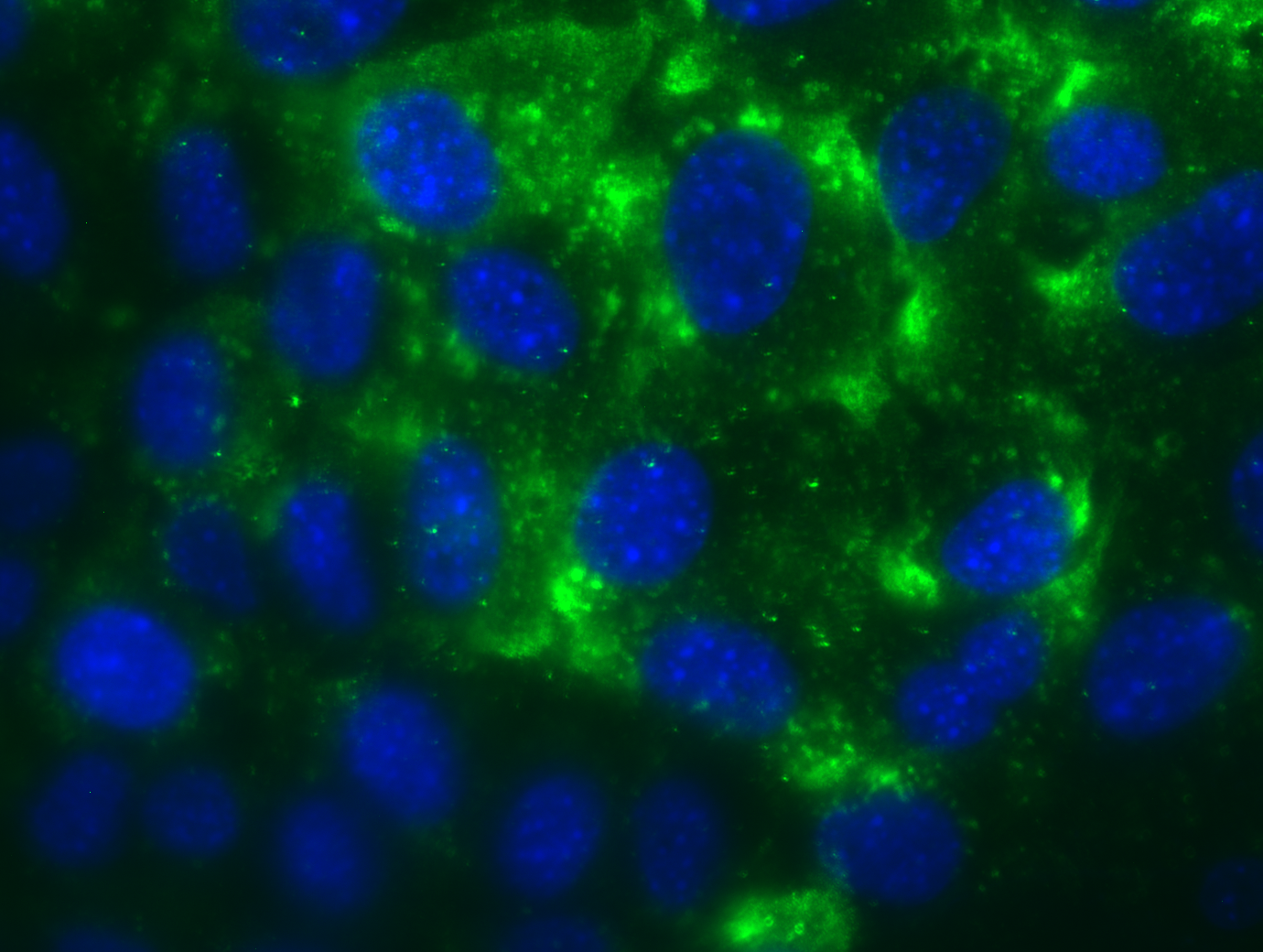

Supplement: Supplementary file 1 — Source Data Fig. 1 [file 44318_2024_53_MOESM1_ESM.zip › SD Figure 1/1D/Perilipin-1_Images/NAA40-KD.TIF]

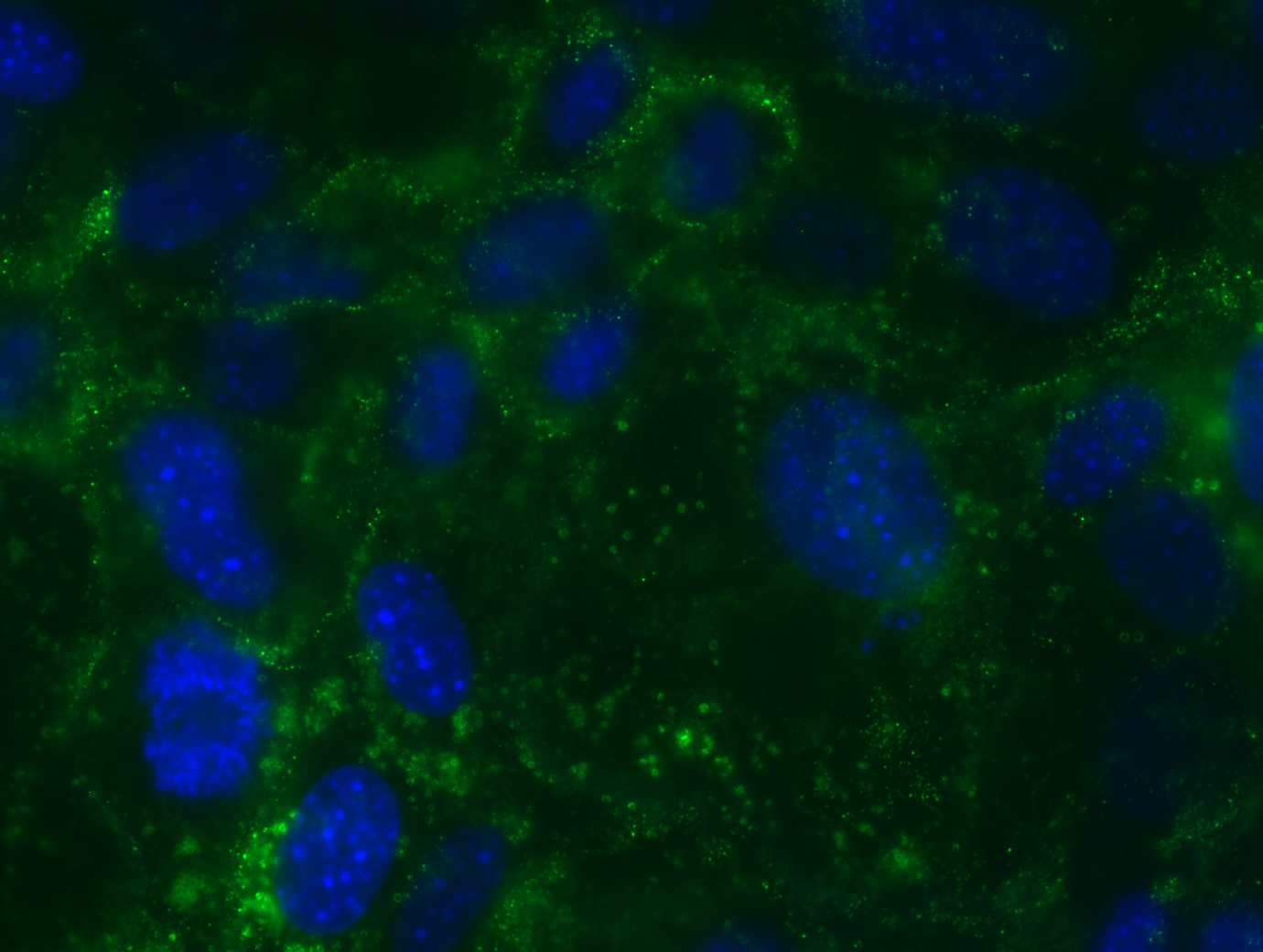

Supplement: Supplementary file 1 — Source Data Fig. 1 [file 44318_2024_53_MOESM1_ESM.zip › SD Figure 1/1D/Perilipin-1_Images/Scramble.TIF]

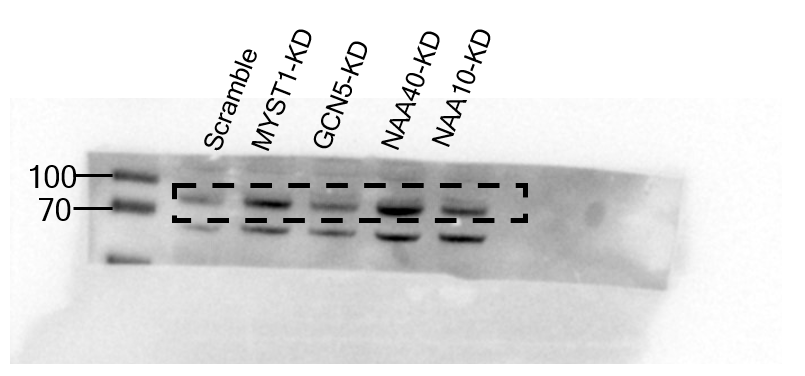

Supplement: Supplementary file 4 — Source Data Fig. 4 [file 44318_2024_53_MOESM4_ESM.zip › SD Figure 4/4B/Western Blots/ACSS2/ACSS2_WB.png]

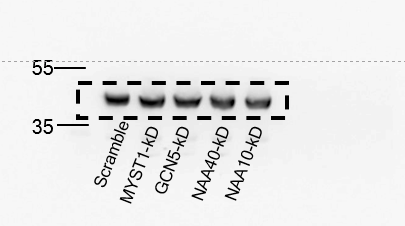

Supplement: Supplementary file 4 — Source Data Fig. 4 [file 44318_2024_53_MOESM4_ESM.zip › SD Figure 4/4B/Western Blots/Actin/Actin.png]

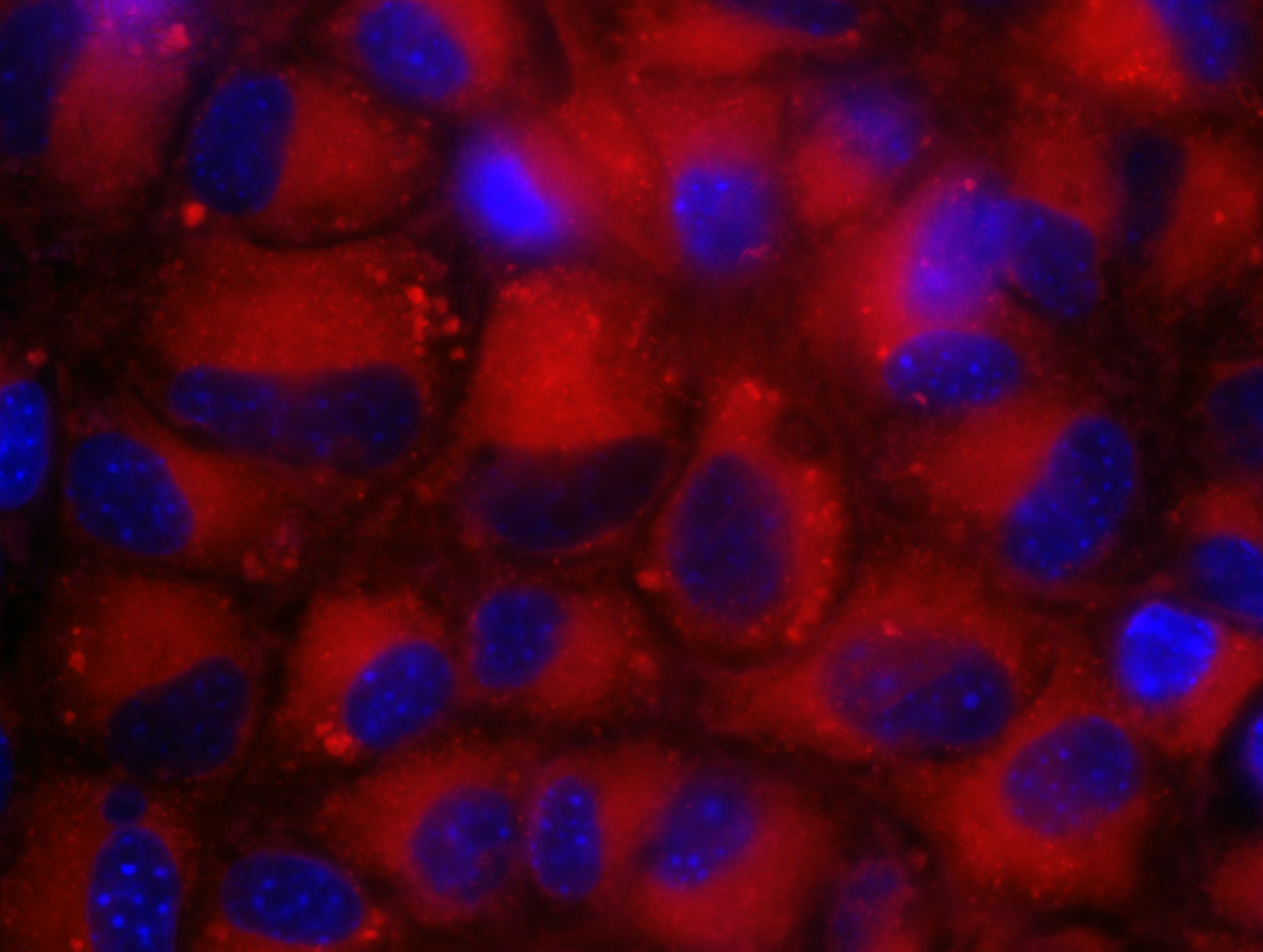

Supplement: Supplementary file 4 — Source Data Fig. 4 [file 44318_2024_53_MOESM4_ESM.zip › SD Figure 4/4E/Nile Red Images/GCN5-KD+ACSS2-KD.tif]

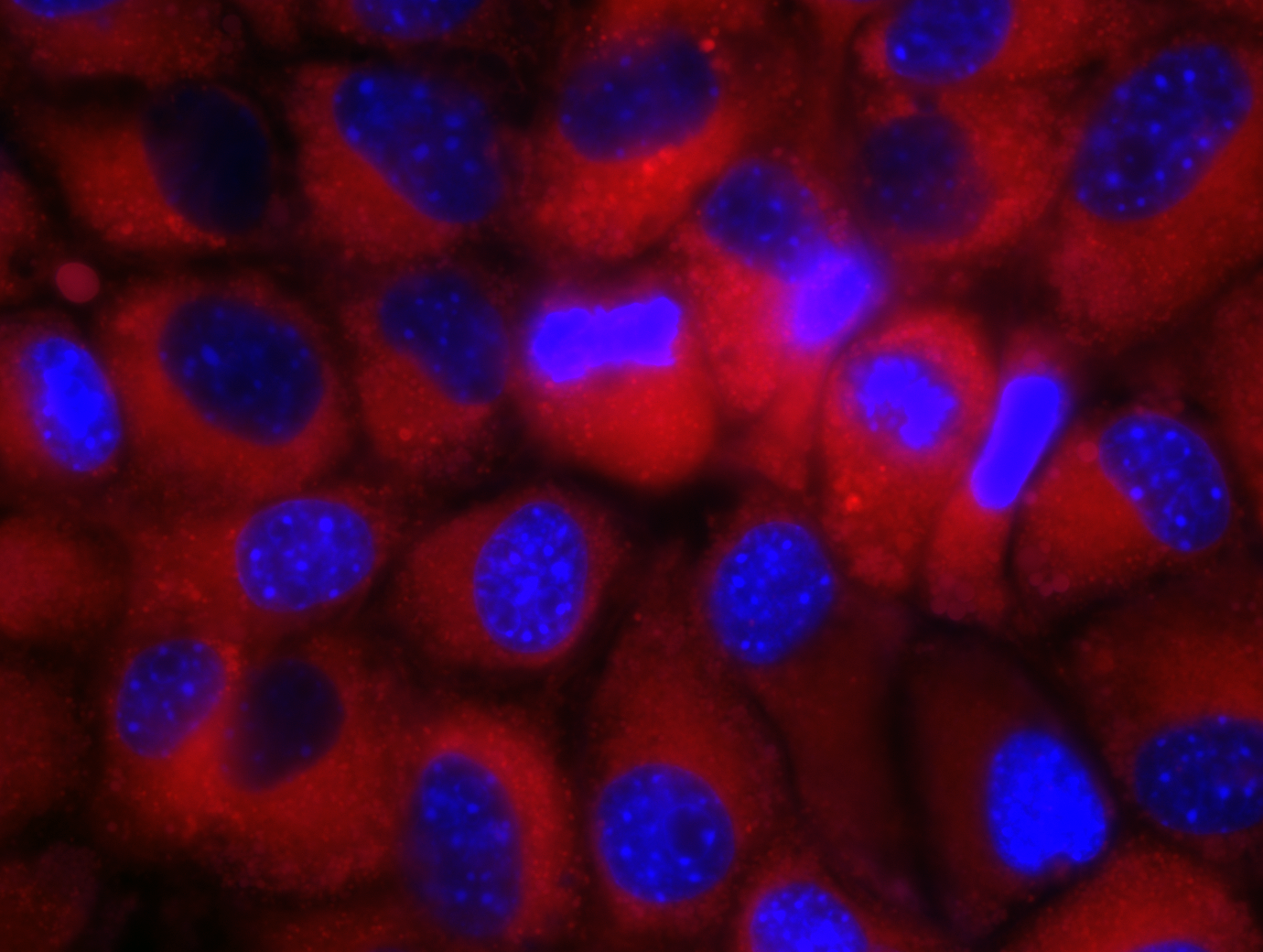

Supplement: Supplementary file 4 — Source Data Fig. 4 [file 44318_2024_53_MOESM4_ESM.zip › SD Figure 4/4E/Nile Red Images/MYST1-KD+ACSS2-KD.tif]

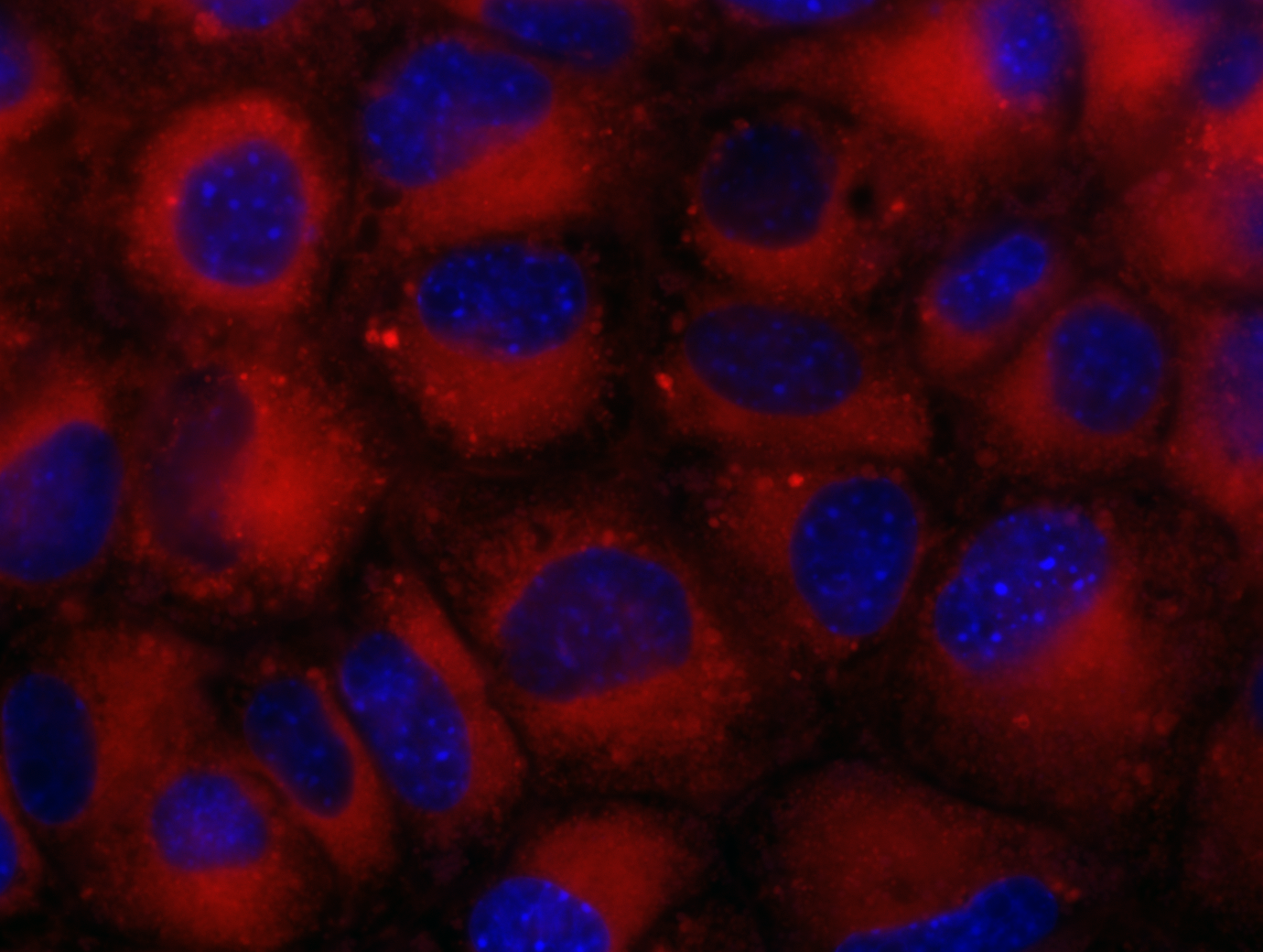

Supplement: Supplementary file 4 — Source Data Fig. 4 [file 44318_2024_53_MOESM4_ESM.zip › SD Figure 4/4E/Nile Red Images/NAA10-KD+ACSS2-KD.tif]

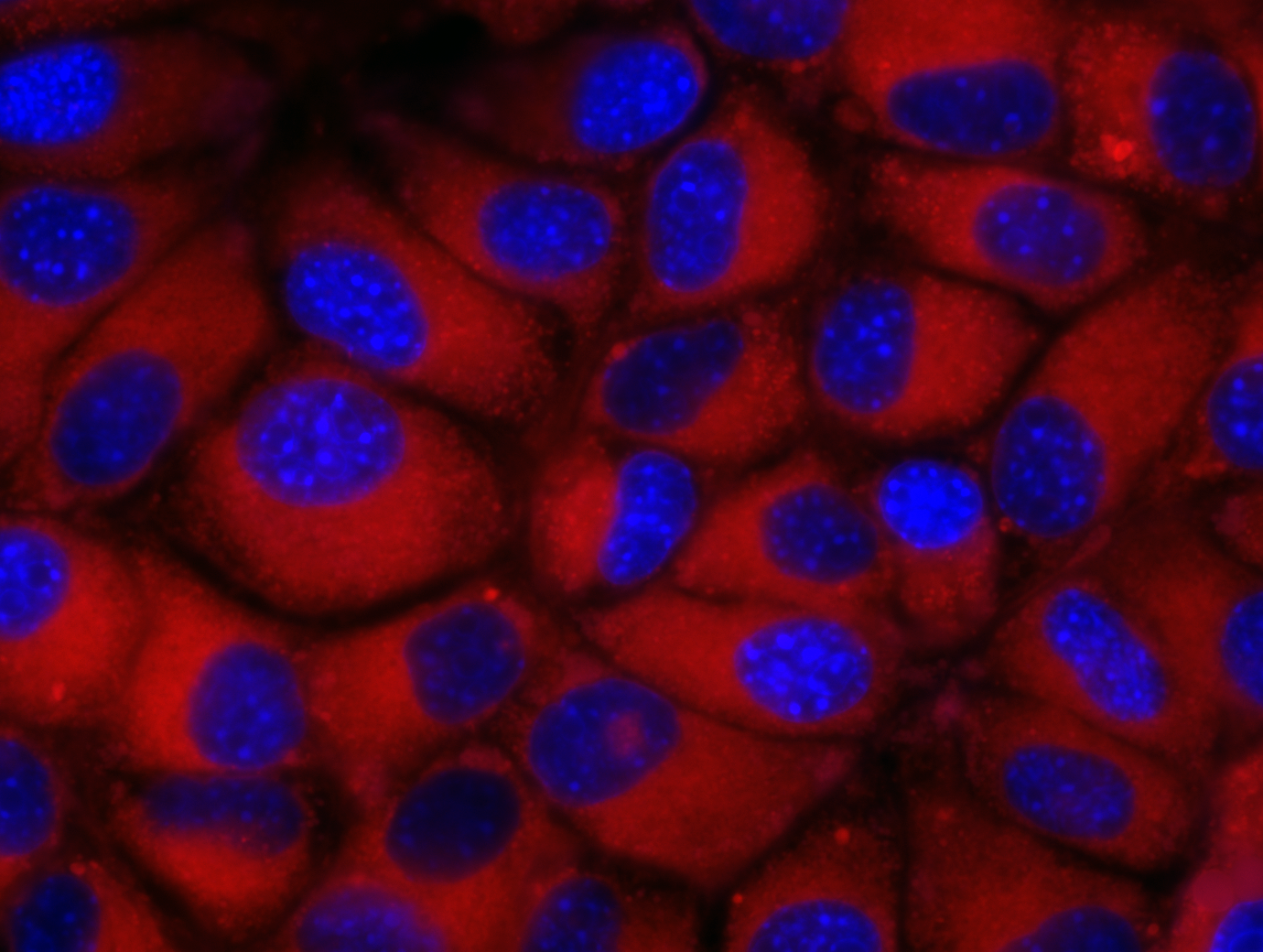

Supplement: Supplementary file 4 — Source Data Fig. 4 [file 44318_2024_53_MOESM4_ESM.zip › SD Figure 4/4E/Nile Red Images/NAA40-KD+ACSS2-KD.tif]

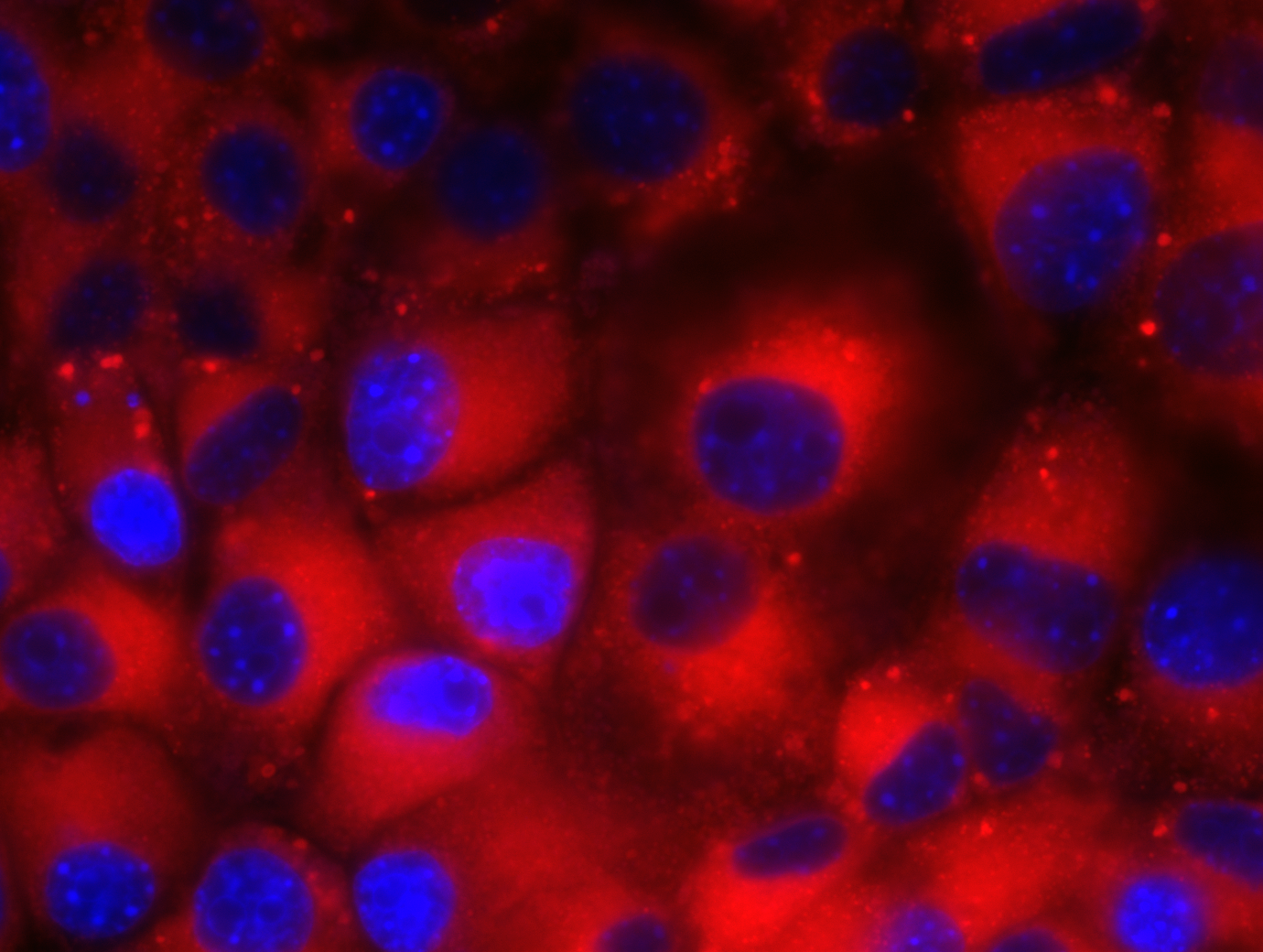

Supplement: Supplementary file 4 — Source Data Fig. 4 [file 44318_2024_53_MOESM4_ESM.zip › SD Figure 4/4E/Nile Red Images/Scramble.tif]

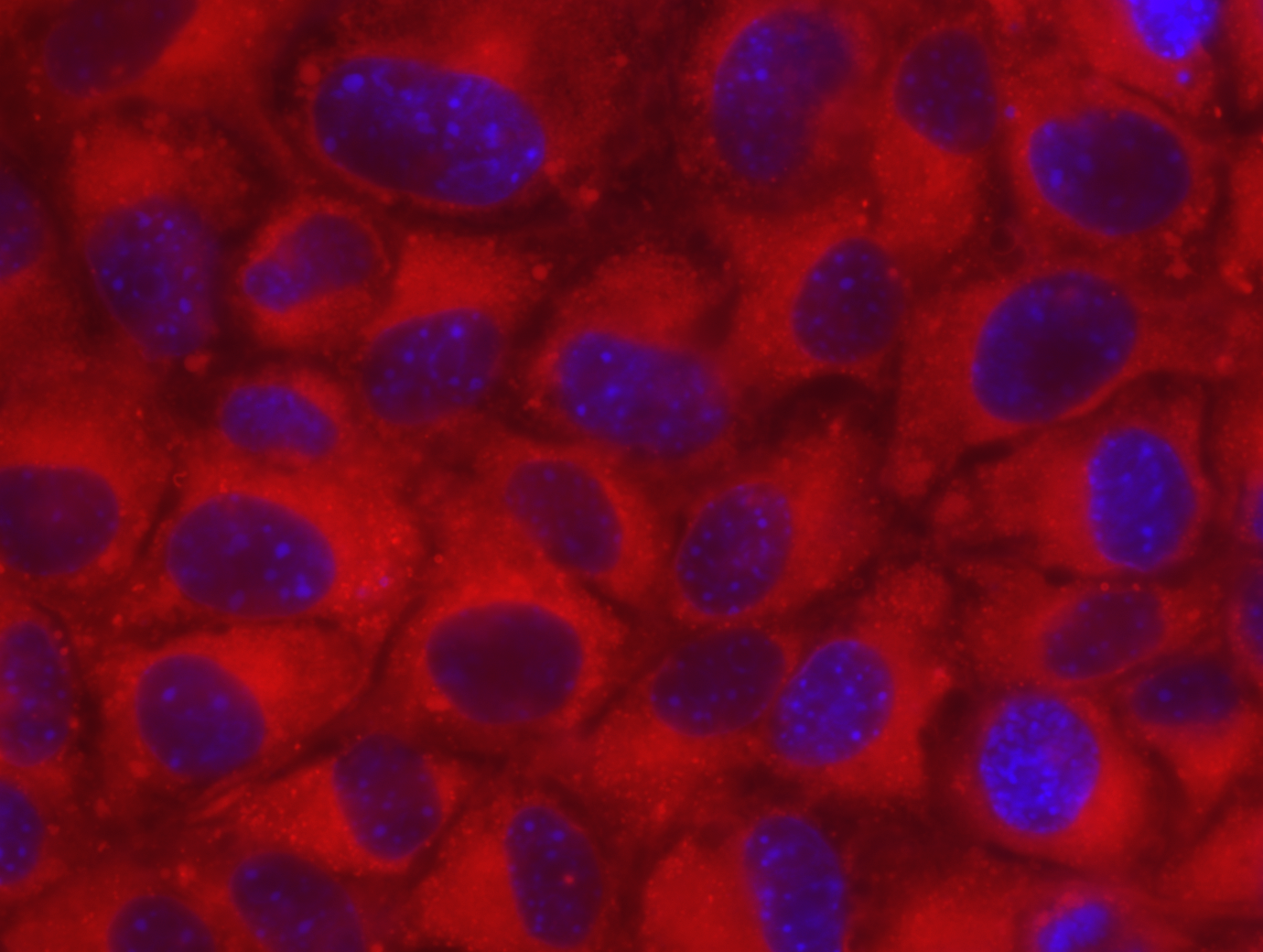

Supplement: Supplementary file 5 — Source Data Fig. 5 [file 44318_2024_53_MOESM5_ESM.zip › SD Figure 5/5B/Nile Red Images/dOE_Control.tif]

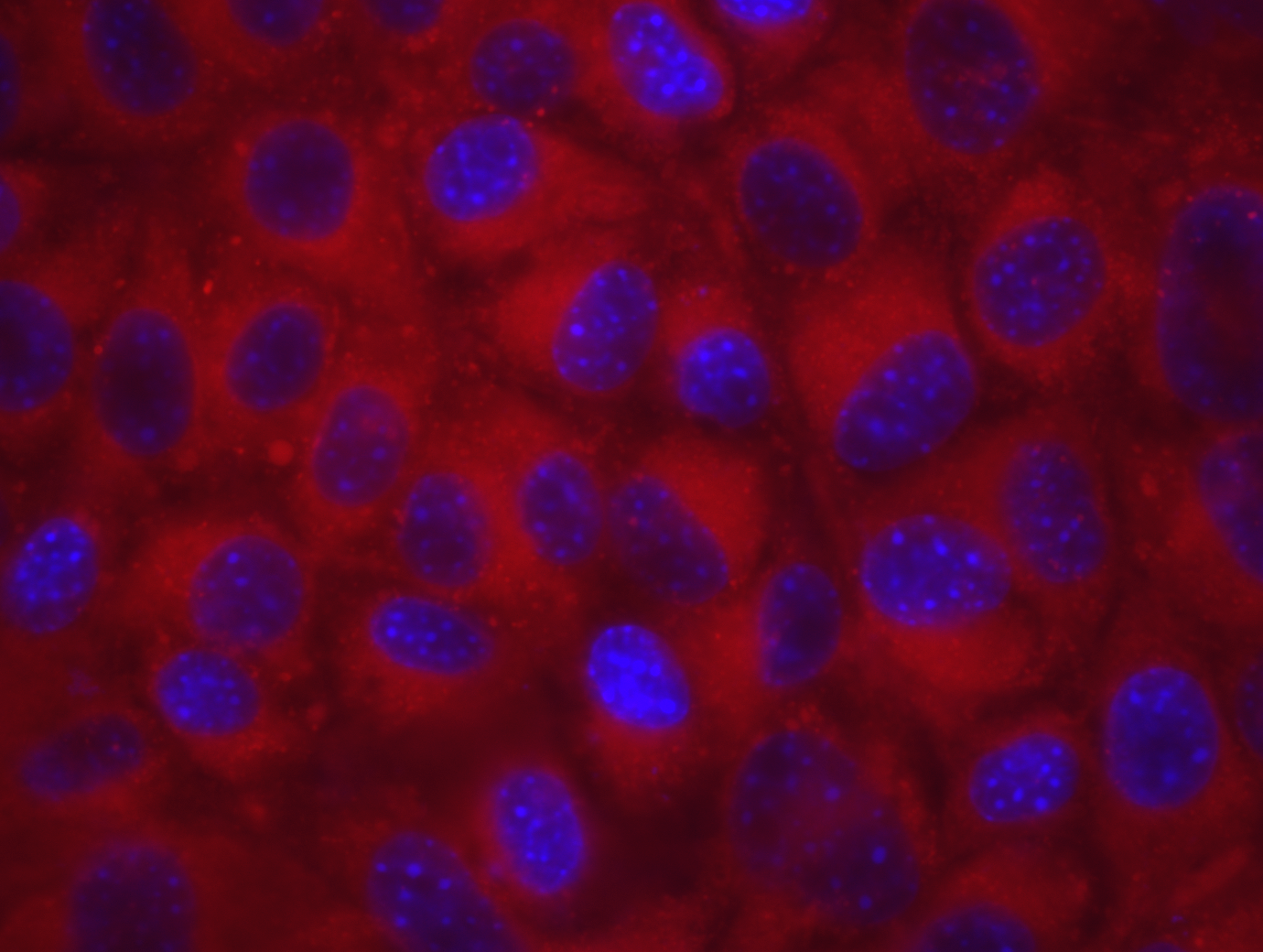

Supplement: Supplementary file 5 — Source Data Fig. 5 [file 44318_2024_53_MOESM5_ESM.zip › SD Figure 5/5B/Nile Red Images/dOE_HFHG.tif]

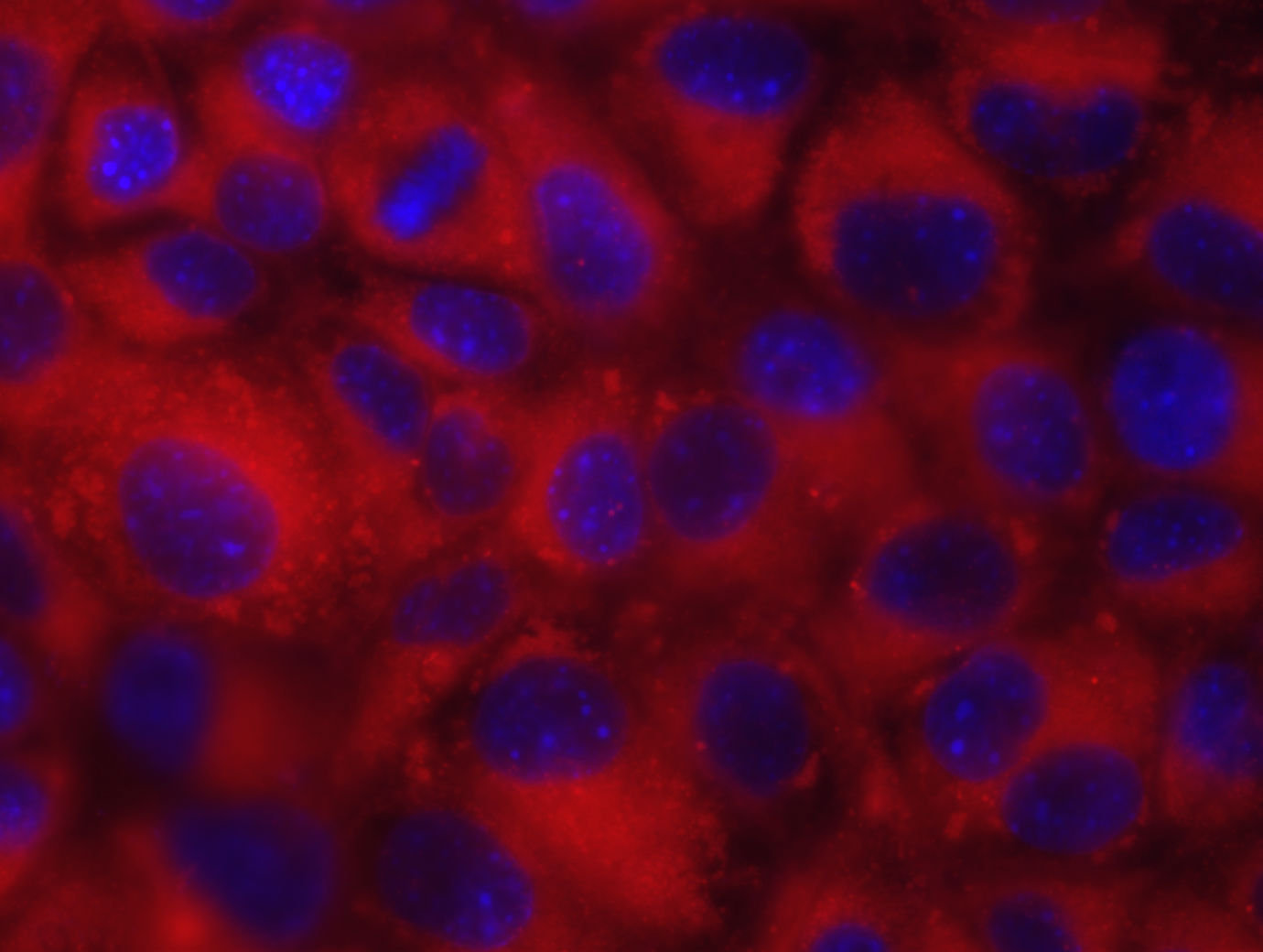

Supplement: Supplementary file 5 — Source Data Fig. 5 [file 44318_2024_53_MOESM5_ESM.zip › SD Figure 5/5B/Nile Red Images/EV_Control.tif]

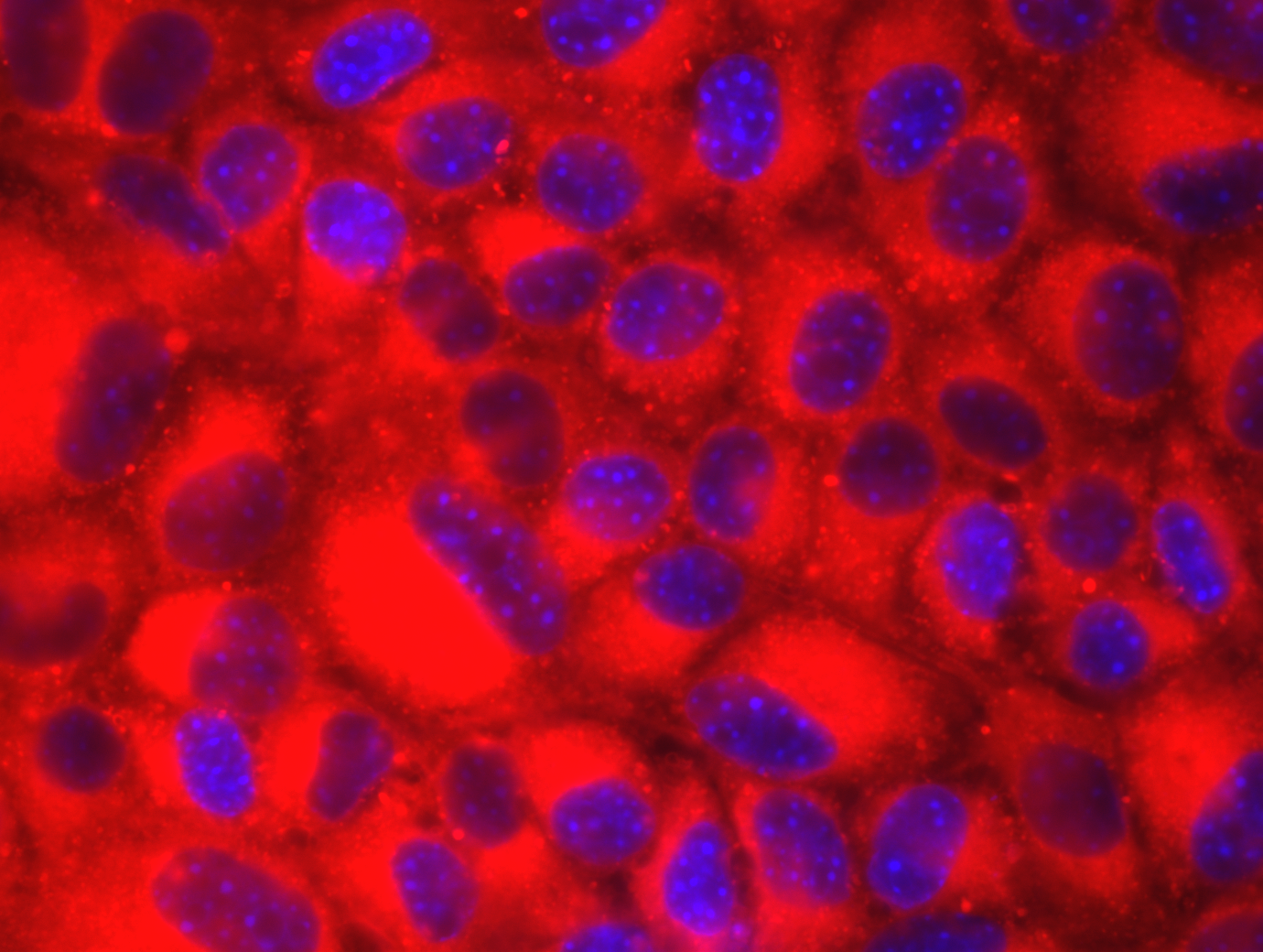

Supplement: Supplementary file 5 — Source Data Fig. 5 [file 44318_2024_53_MOESM5_ESM.zip › SD Figure 5/5B/Nile Red Images/EV_HFHG.tif]

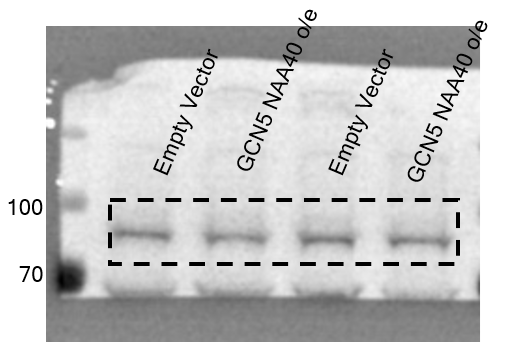

Supplement: Supplementary file 5 — Source Data Fig. 5 [file 44318_2024_53_MOESM5_ESM.zip › SD Figure 5/5E/Western Blots/ACSS2/ACSS2.png]

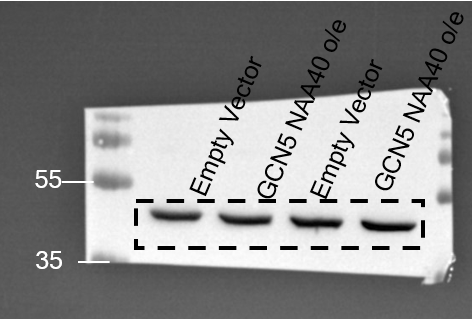

Supplement: Supplementary file 5 — Source Data Fig. 5 [file 44318_2024_53_MOESM5_ESM.zip › SD Figure 5/5E/Western Blots/ACTIN/ACTIN.png]

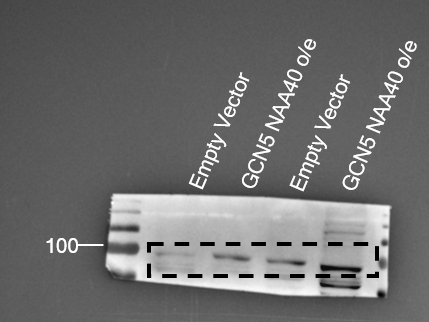

Supplement: Supplementary file 5 — Source Data Fig. 5 [file 44318_2024_53_MOESM5_ESM.zip › SD Figure 5/5E/Western Blots/GCN5/GCN5.png]

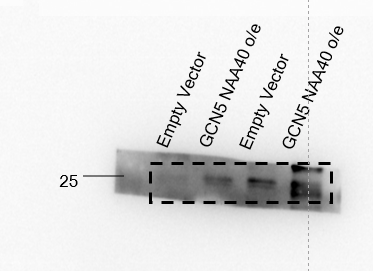

Supplement: Supplementary file 5 — Source Data Fig. 5 [file 44318_2024_53_MOESM5_ESM.zip › SD Figure 5/5E/Western Blots/NAA40/NAA40.png]

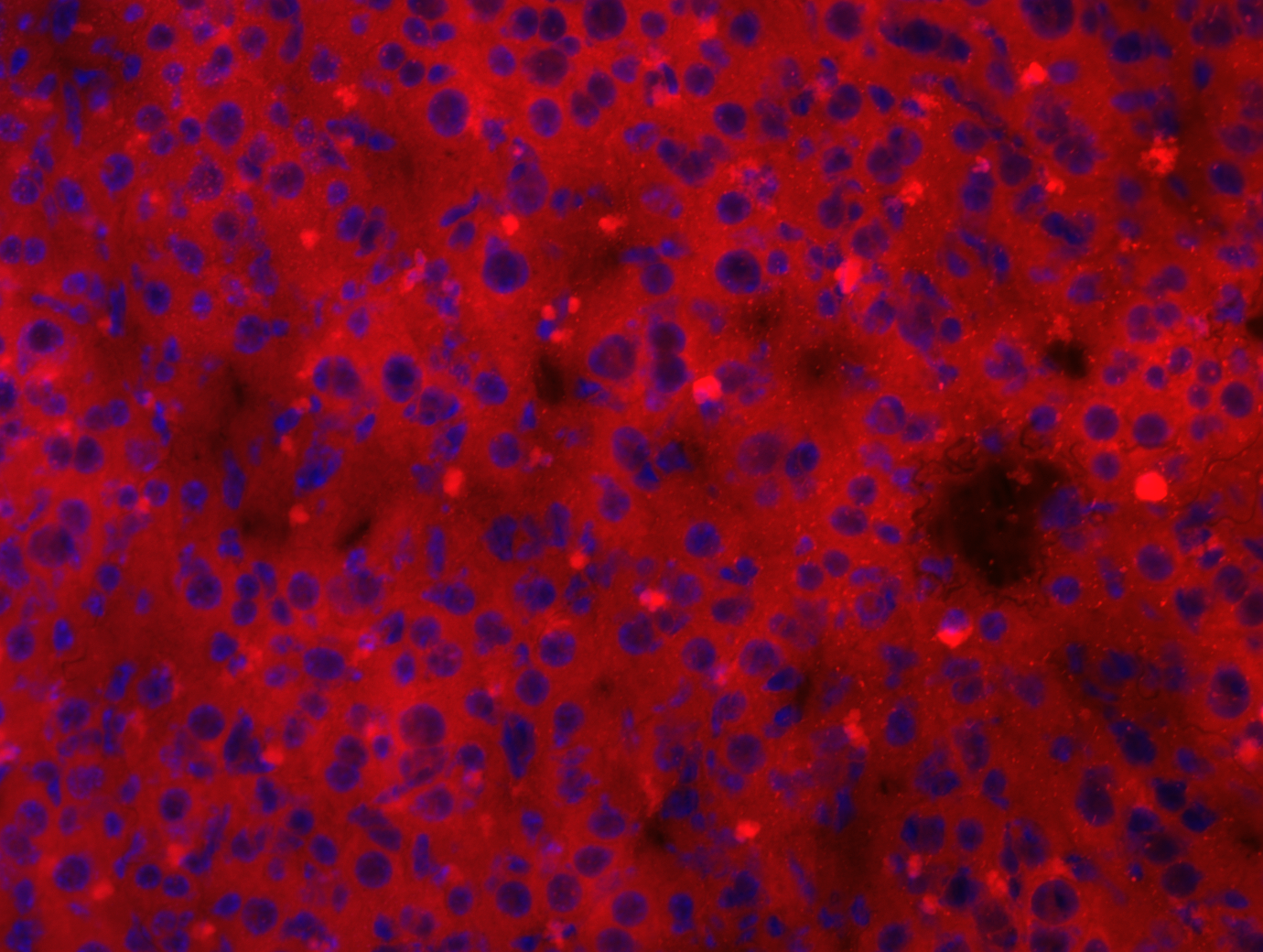

Supplement: Supplementary file 6 — Source Data Fig. 6 [file 44318_2024_53_MOESM6_ESM.zip › SD Figure 6/6D/Nile Red Images/dOE_Control.tif]

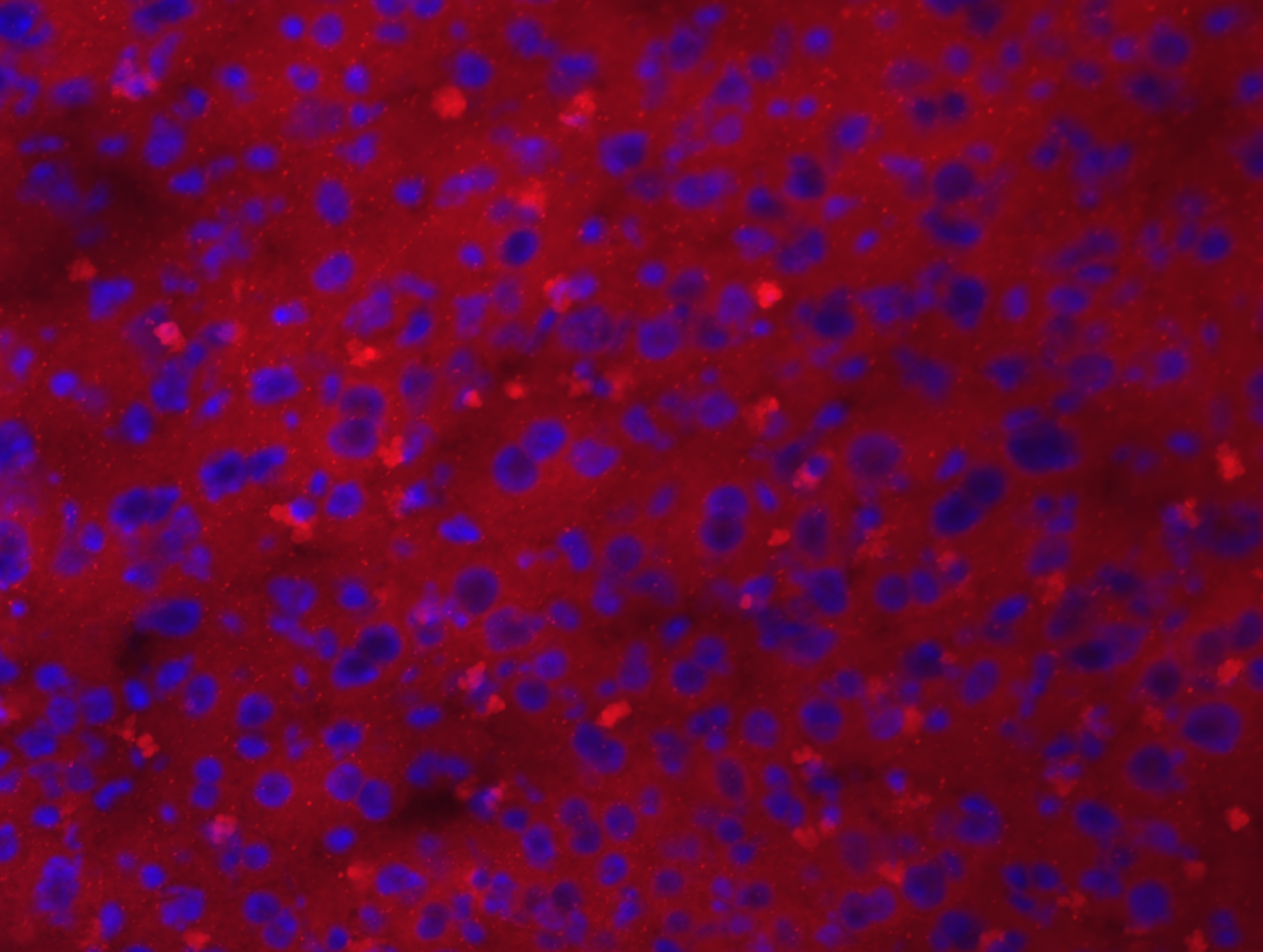

Supplement: Supplementary file 6 — Source Data Fig. 6 [file 44318_2024_53_MOESM6_ESM.zip › SD Figure 6/6D/Nile Red Images/dOE_HFHG.TIF]

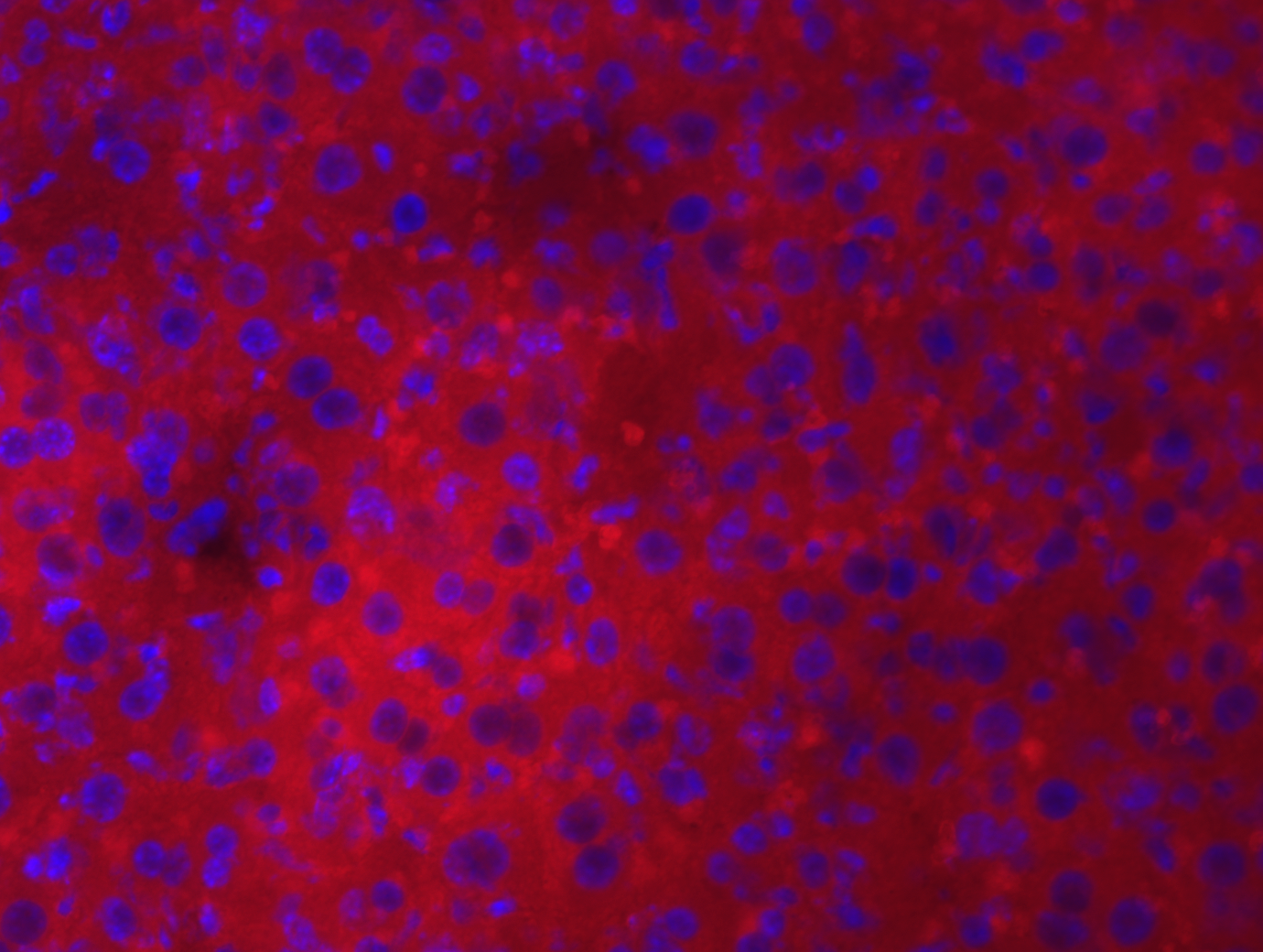

Supplement: Supplementary file 6 — Source Data Fig. 6 [file 44318_2024_53_MOESM6_ESM.zip › SD Figure 6/6D/Nile Red Images/EV_Control.TIF]

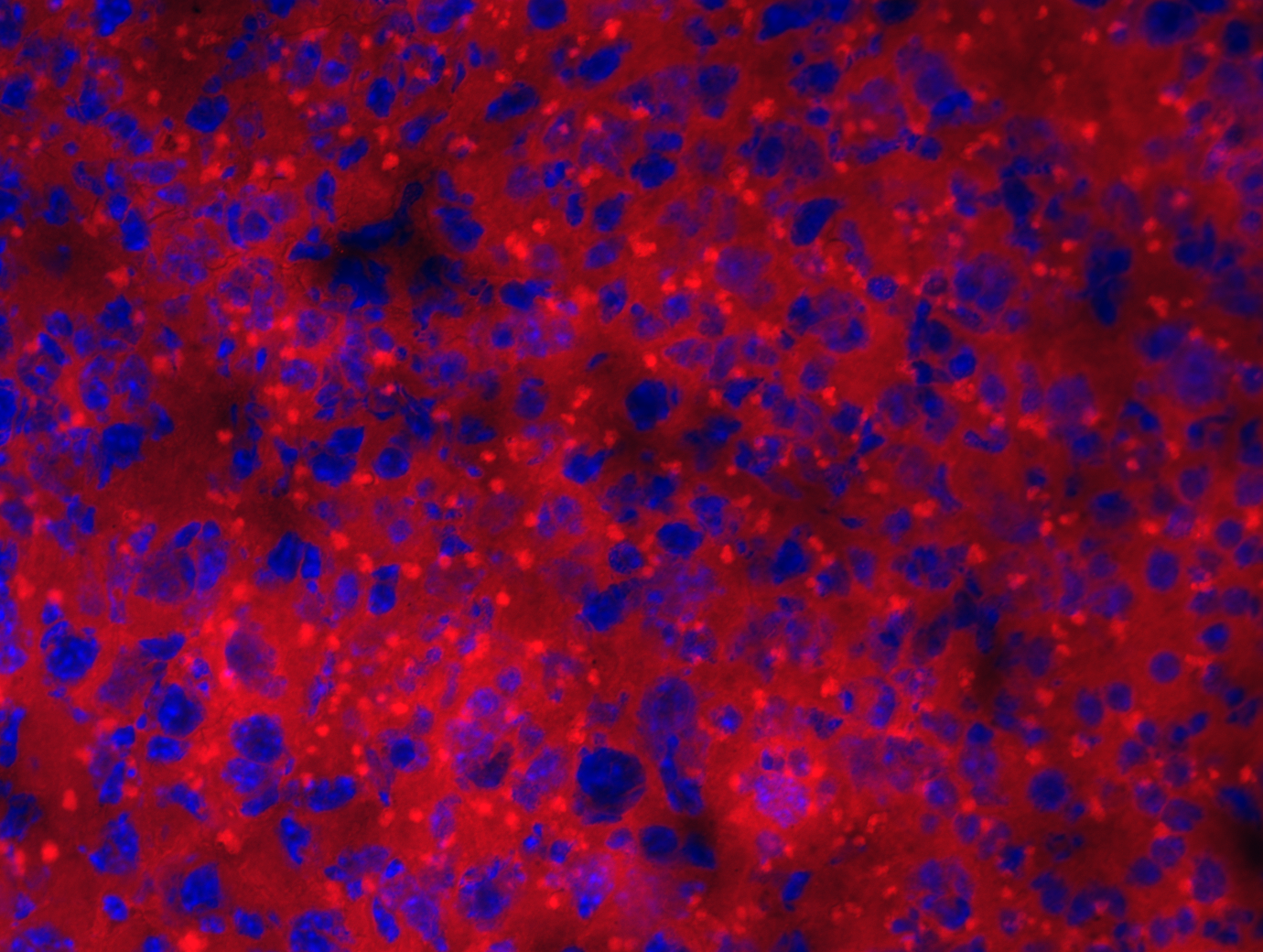

Supplement: Supplementary file 6 — Source Data Fig. 6 [file 44318_2024_53_MOESM6_ESM.zip › SD Figure 6/6D/Nile Red Images/EV_HFHG.tif]

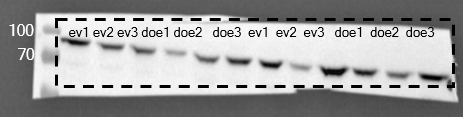

Supplement: Supplementary file 6 — Source Data Fig. 6 [file 44318_2024_53_MOESM6_ESM.zip › SD Figure 6/6G/Western Blots/ACSS2.png]

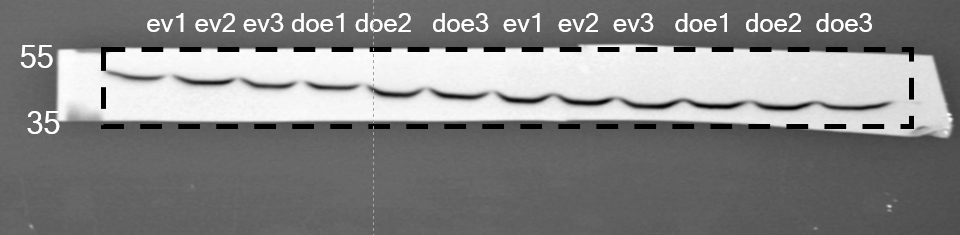

Supplement: Supplementary file 6 — Source Data Fig. 6 [file 44318_2024_53_MOESM6_ESM.zip › SD Figure 6/6G/Western Blots/ACTIN.png]

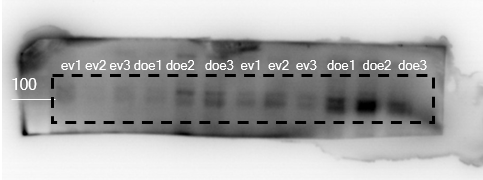

Supplement: Supplementary file 6 — Source Data Fig. 6 [file 44318_2024_53_MOESM6_ESM.zip › SD Figure 6/6G/Western Blots/GCN5.png]

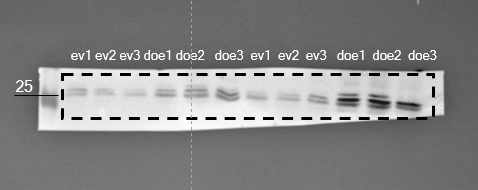

Supplement: Supplementary file 6 — Source Data Fig. 6 [file 44318_2024_53_MOESM6_ESM.zip › SD Figure 6/6G/Western Blots/NAA40.png]

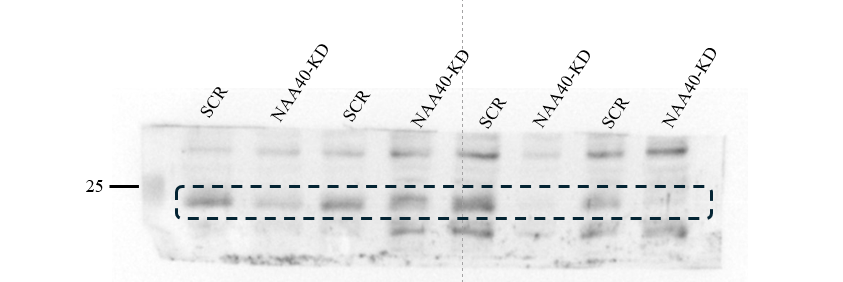

Supplement: Supplementary file 7 — Figure EV3 Source Data [file 44318_2024_53_MOESM7_ESM.zip › SD Figure EV3/3C/NAA40 WB.png]
