# Supplementary material for: Hyperacetylated histone H4 is a source of carbon contributing to lipid synthesis
Source: EMBO J. 2024 Feb 21;43(7):4. doi: 10.1038/s44318-024-00053-0 (PMC10987603; doi:10.1038/s44318-024-00053-0)
Supplement: Supplementary file 9 — Expanded View Figures [file 44318_2024_53_MOESM9_ESM.pdf]

Expanded View Figures

Figure EV1. Acetyltransferase depletion attenuates insulin signalling and does not affect the transcription of genes involved in lipid synthesis.

(**A**) RT-qPCR analysis of expression of *Myst-1*, *Gcn5*, *Naa40*, and *Naa10* mRNA levels in scramble, MYST1-KD, GCN5-KD, NAA40-KD, and NAA10-KD cells, respectively, after 48 h of siRNA treatment. $n = 3$ biological replicates/group. Statistical analysis was performed using two-way ANOVA with post hoc Tukey's multiple-comparisons test; ****$P \leq 0.0001$. (**B**) Relative intensity (normalised to scramble) of indicated metabolites in scramble, MYST1-KD, GCN5-KD, NAA40-KD, and NAA10-KD cells measured by MS after 48 h of siRNA treatment. $n = 3$ biological replicates/group. Statistical analysis was performed using a one-way ANOVA with post hoc Dunnett's multiple-comparisons test. Only P values which are *$P \leq 0.05$ are indicated. All comparisons between KD and Scramble that are non-significant are not indicated. (**C**) Relative intensity (normalised to scramble) of free fatty acids and cholesterol in scramble, MYST1-KD, GCN5-KD, NAA40-KD, and NAA10-KD cells measured by MS. $n = 3$ biological replicates/group. Statistical analysis was performed using a one-way ANOVA with post hoc Dunnett's multiple-comparisons test; ns non-significant. (**D**) Representative immunoblots of phopshoAKT ser473, totalAKT and β-actin in scramble, MYST1-KD, GCN5-KD, NAA40-KD cells, and NAA10-KD respectively, after 48 h of siRNA treatment. $n = 3$ biological replicates/group. (**E**) Mean fluorescent uptake quantification by FACS in scramble, MYST1-KD, GCN5-KD, NAA40-KD cells, and NAA10-KD, respectively, after 48 h of siRNA treatment. $n = 3$ biological replicates/group. Statistical analysis was performed using a one-way ANOVA with post hoc Dunnett's multiple-comparisons test; **$P \leq 0.01$, ns non-significant. (**F**) RT-qPCR analysis of expression of *Acly*, *Fasn*, *Sbrebf*, mRNA levels in scramble, MYST1-KD, GCN5-KD, NAA40-KD, and NAA10-KD cells, respectively, after 48 h of siRNA treatment. $n = 3$ biological replicates/group. Statistical analysis was performed using a two-way ANOVA with post hoc Tukey's multiple-comparisons test; ns non-significant. (**G**) Volcano plot comparing mRNA levels between NAA40-KD and SCR control cells as determined by RNA-seq analysis after 12 h of siRNA treatment. Upregulated genes upon loss of NAA40 are shown in red (adjusted $P < 0.05$ and logFC > 0.5) and downregulated genes in blue (adjusted $P < 0.05$ and logFC < −0.5). $n = 3$ biological replicates/group. Statistical analysis was performed by a paired T test and corrected with the False Discovery Rate (FDR). (**H**) Volcano plot comparing mRNA levels between NAA40-KD and SCR control cells as determined by RNA-seq analysis after 48 h of siRNA treatment. Upregulated genes upon loss of NAA40 are shown in red (adjusted $P < 0.05$ and logFC > 0.5) and downregulated genes in blue (adjusted $P < 0.05$ and logFC < −0.5). $n = 3$ biological replicates/group. Statistical analysis was performed by a paired T test and corrected with the False Discovery Rate (FDR). (**I**) Gene ontology analysis of all differentially expressed genes showing enriched biological processes following depletion of NAA40 after 48 h of siRNA treatment. $n = 3$ biological replicates/group. Data information: all data are presented as mean ± SEM.

 © The Author(s)

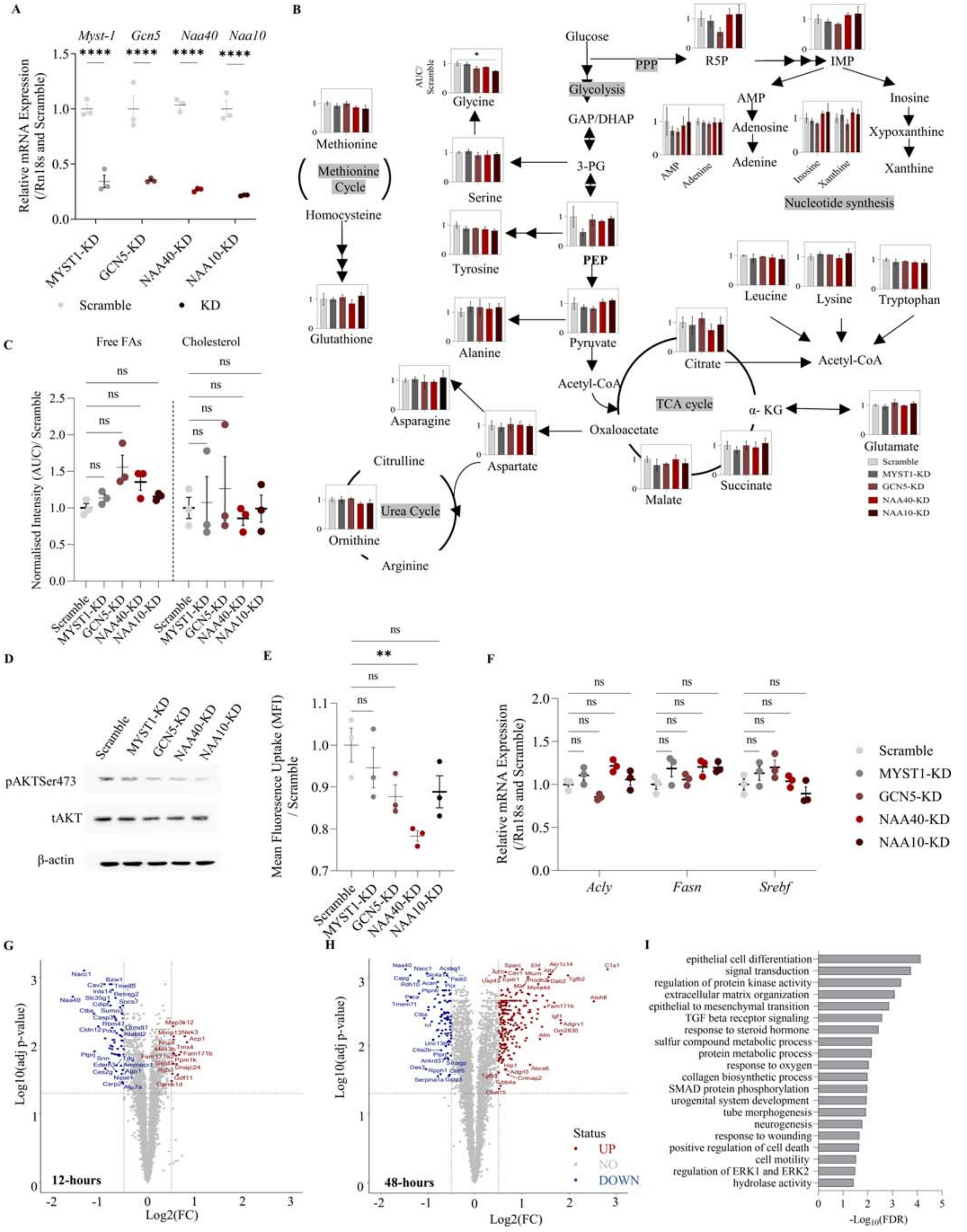

A
Myst-1
Gcn5
Naa40
Naa10
Relative mRNA Expression (/Rn18s and Scramble)
MYST1-KD
GCN5-KD
NAA40-KD
NAA10-KD
Scramble
KD
B
Glucose
PPP
R5P
IMP
Glycolysis
GAP/DHAP
3-PG
PEP
Pyruvate
Acetyl-CoA
AMP
Adenosine
Adenine
Inosine
Xypoxanthine
Xanthine
Nucleotide synthesis
AUC/ Scramble
Glycine
Methionine
Methionine Cycle
Homocysteine
Glutathione
Serine
Tyrosine
Alanine
Leucine
Lysine
Tryptophan
Acetyl-CoA
Citrate
TCA cycle
α- KG
Glutamate
Oxaloacetate
Asparagine
Aspartate
Malate
Succinate
Citrulline
Urea Cycle
Ornithine
Arginine
Scramble
MYST1-KD
GCN5-KD
NAA40-KD
NAA10-KD
C
Free FAs
Cholesterol
Normalised Intensity (AUC)/ Scramble
D
pAKTSer473
tAKT
β-actin
E
Mean Fluorescence Uptake (MFI) / Scramble
F
Relative mRNA Expression (/Rn18s and Scramble)
Acly
Fasn
Srebf
G
Log10(adj p-value)
Log2(FC)
12-hours
H
48-hours
Status
UP
NO
DOWN
I
epithelial cell differentiation
signal transduction
regulation of protein kinase activity
extracellular matrix organization
epithelial to mesenchymal transition
TGF beta receptor signaling
response to steroid hormone
sulfur compound metabolic process
protein metabolic process
response to oxygen
collagen biosynthetic process
SMAD protein phosphorylation
urogenital system development
tube morphogenesis
neurogenesis
response to wounding
positive regulation of cell death
cell motility
regulation of ERK1 and ERK2
hydrolase activity
-Log10(FDR)

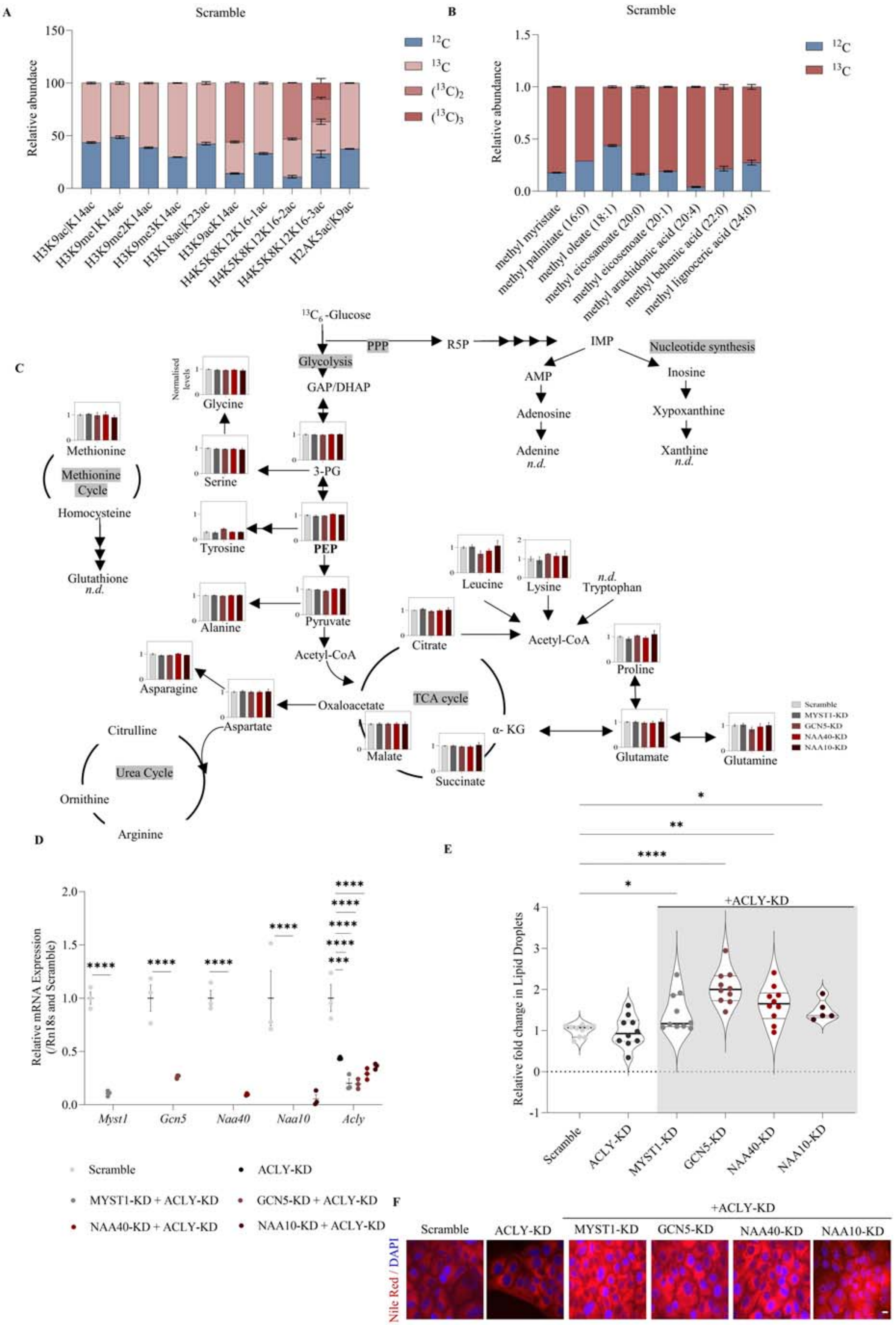
A
Scramble
Relative abundace
150
100
50
0
H3K9ac|K14ac
H3K9me1K14ac
H3K9me2K14ac
H3K9me3K14ac
H3K18ac|K23ac
H3K9acK14ac
H4K5K8K12K16-1ac
H4K5K8K12K16-2ac
H4K5K8K12K16-3ac
H2AK5ac|K9ac
12C
13C
(13C)2
(13C)3
B
Scramble
Relative abundance
1.5
1.0
0.5
0.0
methyl myristate
methyl palmitate (16:0)
methyl oleate (18:1)
methyl eicosanoate (20:0)
methyl eicosenoate (20:1)
methyl arachidonic acid (20:4)
methyl behenic acid (22:0)
methyl lignoceric acid (24:0)
12C
13C
C
13C6-Glucose
PPP
R5P
IMP
Nucleotide synthesis
Glycolysis
GAP/DHAP
AMP
Adenosine
Adenine
n.d.
Inosine
Xypoxanthine
Xanthine
n.d.
Normalised levels
Glycine
Methionine
Methionine Cycle
Homocysteine
Glutathione
n.d.
Serine
3-PG
Tyrosine
PEP
Alanine
Pyruvate
Acetyl-CoA
Leucine
Lysine
n.d.
Tryptophan
Citrate
Acetyl-CoA
Proline
Asparagine
Aspartate
Oxaloacetate
TCA cycle
α- KG
Malate
Succinate
Glutamate
Glutamine
Scramble
MYST1-KD
GCN5-KD
NAA40-KD
NAA10-KD
Citrulline
Urea Cycle
Ornithine
Arginine
D
Relative mRNA Expression (/Rn18s and Scramble)
2.0
1.5
1.0
0.5
0.0
Myst1
Gcn5
Naa40
Naa10
Acly
Scramble
ACLY-KD
MYST1-KD + ACLY-KD
GCN5-KD + ACLY-KD
NAA40-KD + ACLY-KD
NAA10-KD + ACLY-KD
E
Relative fold change in Lipid Droplets
+ACLY-KD
Scramble
ACLY-KD
MYST1-KD
GCN5-KD
NAA40-KD
NAA10-KD
F
Scramble
ACLY-KD
+ACLY-KD
MYST1-KD
GCN5-KD
NAA40-KD
NAA10-KD
Nile Red / DAPI

 © The Author(s)

Figure EV2. Lipid synthesis upon acetyltransferase depletion is not associated with ACLY in AML12 hepatocytes.

(**A**) Relative abundance of ^{12}C and ^{13}C in histone acetylation peptides measured by MS in scramble cells 48 h after siRNA treatment and supplementation with $^{13}C_6$-Glucose. $n = 3$ biological replicates/group. (**B**) Relative abundance of ^{12}C and ^{13}C methyl fatty acids measured by MS in scramble cells 48 h after siRNA treatment and supplementation with $^{13}C_6$-Glucose. $n = 3$ biological replicates/group. (**C**) Normalised levels (% label/ scramble) incorporation in indicated metabolites in scramble, MYST1-KD, GCN5-KD, NAA40-KD, and NAA10-KD cells measured by MS, 48 h after siRNA treatment. $n = 3$ biological replicates/group. Statistical analysis was performed using a one-way ANOVA with post hoc Dunnett's multiple-comparisons test. All comparisons between KD and Scramble that are non-significant are not indicated. (**D**) RT-qPCR analysis of *Myst-1*, *Gcn5*, *Naa40, Naa10* and *Acly* mRNA levels in scramble, ACLY-KD and the indicated double-KD cells after 48 h of siRNA treatment. $n = 3$ biological replicates/group. Statistical analysis was performed using two-way ANOVA with post hoc Tukey's multiple-comparisons test; ***$P \le 0.001$, ****$P \le 0.0001$. (**E**) Quantification of relative lipid droplets in scramble, ACLY-KD and the indicated double-KD cells after 48 h of siRNA treatment. $n = 6$–8 biological replicates/group. Statistical analysis was performed using a one-way ANOVA with post hoc Dunnett's multiple-comparisons test; *$P \le 0.05$, **$P \le 0.01$, ****$P \le 0.0001$. (**F**) Representative images of lipid droplets Nile red (red) and nuclei by DAPI (blue) of scramble, ACLY-KD and the indicated double-KD cells after 48 h of siRNA treatment. Scale bar = 25 µm. $n = 6$–8 biological replicates/group. Data information: (**A–D**) are presented as mean ± SEM; (**E**) is presented as a violin plot.

© The Author(s)

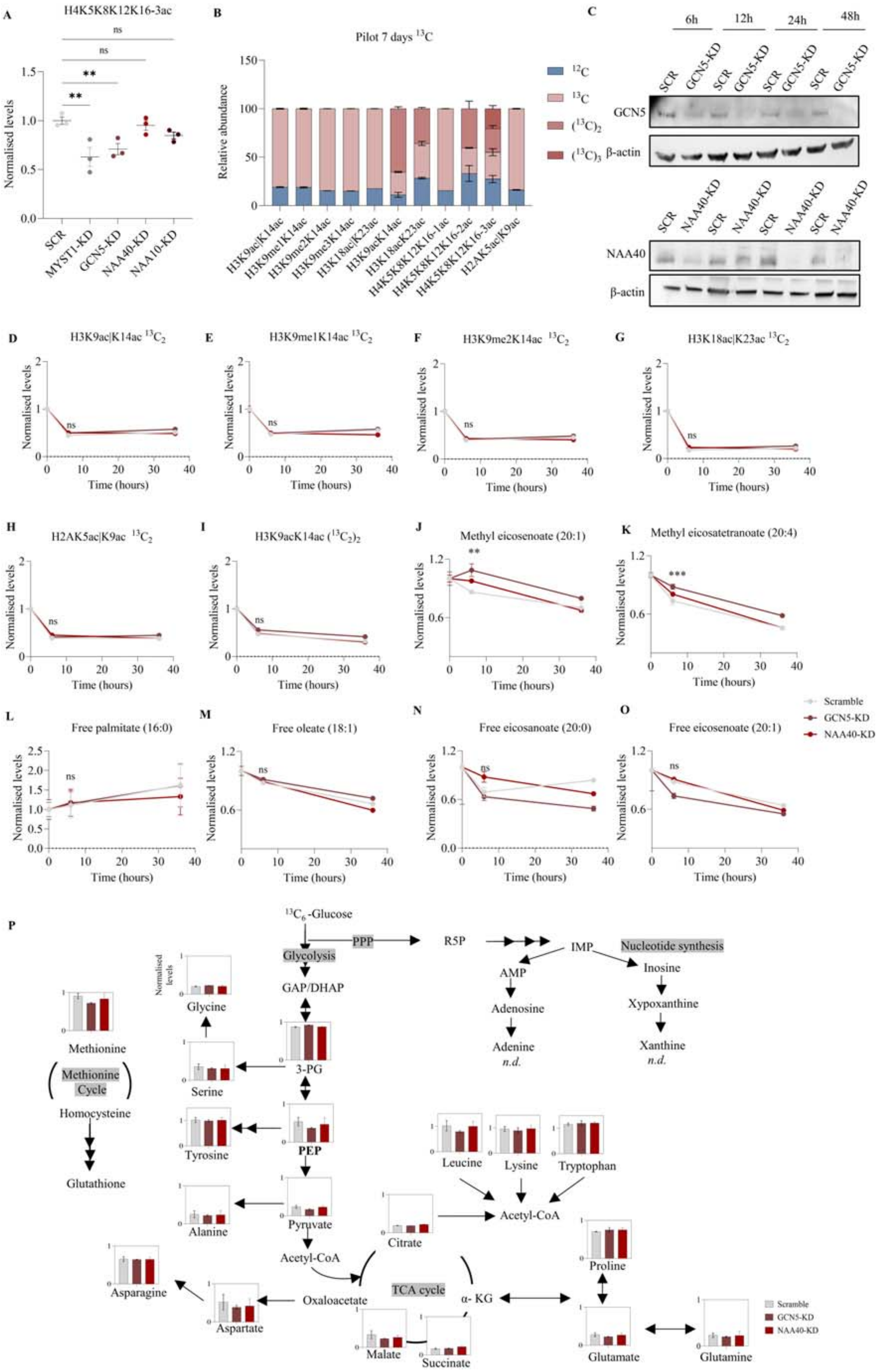

A
H4K5K8K12K16-3ac
Normalised levels
SCR
MYST1-KD
GCN5-KD
NAA40-KD
NAA10-KD
B
Pilot 7 days 13C
Relative abundance
12C
13C
(13C)2
(13C)3
H3K9ac|K14ac
H3K9me1K14ac
H3K9me2K14ac
H3K9me3K14ac
H3K18ac|K23ac
H3K9acK14ac
H3K18acK23ac
H4K5K8K12K16-1ac
H4K5K8K12K16-2ac
H4K5K8K12K16-3ac
H2AK5ac|K9ac
C
6h
12h
24h
48h
SCR
GCN5-KD
GCN5
β-actin
NAA40-KD
NAA40
D
H3K9ac|K14ac 13C2
E
H3K9me1K14ac 13C2
F
H3K9me2K14ac 13C2
G
H3K18ac|K23ac 13C2
H
H2AK5ac|K9ac 13C2
I
H3K9acK14ac (13C2)2
J
Methyl eicosenoate (20:1)
K
Methyl eicosatetranoate (20:4)
L
Free palmitate (16:0)
M
Free oleate (18:1)
N
Free eicosanoate (20:0)
O
Free eicosenoate (20:1)
Time (hours)
Scramble
GCN5-KD
NAA40-KD
P
13C6-Glucose
PPP
R5P
IMP
Nucleotide synthesis
Glycolysis
GAP/DHAP
AMP
Adenosine
Adenine
n.d.
Inosine
Xypoxanthine
Xanthine
n.d.
Glycine
Methionine
Methionine Cycle
Homocysteine
Glutathione
Serine
3-PG
Tyrosine
PEP
Leucine
Lysine
Tryptophan
Alanine
Pyruvate
Citrate
Acetyl-CoA
Proline
Asparagine
Aspartate
Oxaloacetate
TCA cycle
α- KG
Malate
Succinate
Glutamate
Glutamine

© The Author(s)

Figure EV3. Analysis of histone acetylation marks, methyl fatty acids, free fatty acids and aqueous metabolites upon acetyltransferase depletion in chromatin-enriched ^{13}C-labelled cells.

(**A**) Normalised levels (%RA/scramble) of the triply acetylated H4K5K8K12K16 peptide in scramble, MYST1-KD, GCN5-KD, NAA40-KD, and NAA10-KD cells measured by MS, 48 h after siRNA treatment. $n = 3$ biological replicates/group. Statistical analysis was performed using a one-way ANOVA with post hoc Dunnett's multiple-comparisons test; $^{**}P \le 0.01$, ns non-significant. (**B**) Relative abundance of ^{12}C and ^{13}C in acetylated histone peptides in AML12 cells 7 days after supplementation with $^{13}C_6$-Glucose. $n = 3$ biological replicates/group. (**C**) Representative immunoblots of GCN5, NAA40 and β-actin in scramble, GCN5-KD, and NAA40-KD cells at 6, 12, 24 and 48 h after siRNA treatment. $n = 3$ biological replicates/group. (**D**) Normalised levels (%label 6 h/%label 0 h) of singly labelled H3K9ac|K14ac in scramble, GCN5-KD, and NAA40-KD cells. $n = 3$ biological replicates/group. Statistical analysis was performed using two-way ANOVA with post hoc Tukey's multiple-comparisons test; ns non-significant. (**E**) Normalised levels (%label 6 h/%label 0 h) of singly labelled H3K9me1K14ac in scramble, GCN5-KD, and NAA40-KD cells. $n = 3$ biological replicates/group. Statistical analysis was performed using two-way ANOVA with post hoc Tukey's multiple-comparisons test; ns non-significant. (**F**) Normalised levels (%label 6 h/%label 0 h) of singly labelled H3K9me2K14ac in scramble, GCN5-KD, and NAA40-KD cells. $n = 3$ biological replicates/group. Statistical analysis was performed using two-way ANOVA with post hoc Tukey's multiple-comparisons test; ns non-significant. (**G**) Normalised levels (%label 6 h/%label 0 h) of singly labelled H3K18ac | K23 in scramble, GCN5-KD, and NAA40-KD cells. $n = 3$ biological replicates/group. Statistical analysis was performed using two-way ANOVA with post hoc Tukey's multiple-comparisons test; ns non-significant. (**H**) Normalised levels (%label 6 h/%label 0 h) of singly labelled H2AK5ac|K9ac in scramble, GCN5-KD, and NAA40-KD cells. $n = 3$ biological replicates/group. Statistical analysis was performed using two-way ANOVA with post hoc Tukey's multiple-comparisons test; ns non-significant. (**I**) Normalised levels (%label 6 h/%label 0 h) of doubly labelled H3K9acK14ac in scramble, GCN5-KD, and NAA40-KD cells in scramble, GCN5-KD, and NAA40-KD cells. $n = 3$ biological replicates/group. Statistical analysis was performed using two-way ANOVA with post hoc Tukey's multiple-comparisons test; ns non-significant. (**J**) Normalised levels (%label 6 h/%label 0 h) of methyl eicosenoate (20:1) in scramble, GCN5-KD, and NAA40-KD cells. $n = 3$ biological replicates/group. Statistical analysis was performed using two-way ANOVA with post hoc Tukey's multiple-comparisons test; $^{**}P \le 0.01$. (**K**) Normalised levels (%label 6 h/%label 0 h) of Methyl eicosatetranoate (20:4) in scramble, GCN5-KD, and NAA40-KD cells. $n = 3$ biological replicates/group. Statistical analysis was performed using two-way ANOVA with post hoc Tukey's multiple-comparisons test; $^{***}P \le 0.001$. (**L**) Normalised levels (%label 6 h/%label 0 h) of free palmitate (16:0) in scramble, GCN5-KD, and NAA40-KD cells. $n = 3$ biological replicates/group. Statistical analysis was performed using two-way ANOVA with post hoc Tukey's multiple-comparisons test; ns non-significant. (**M**) Normalised levels (%label 6 h/%label 0 h) of free oleate (18:1) in scramble, GCN5-KD, and NAA40-KD cells. $n = 3$ biological replicates/group. Statistical analysis was performed using two-way ANOVA with post hoc Tukey's multiple-comparisons test; ns non-significant. (**N**) Normalised levels (%label 6 h/%label 0 h) of free eicosanoate (20:0) in scramble, GCN5-KD, and NAA40-KD cells. $n = 3$ biological replicates/group. Statistical analysis was performed using two-way ANOVA with post hoc Tukey's multiple-comparisons test; ns non-significant. (**O**) Normalised levels (%label 6 h/%label 0 h) of free eicosenoate (20:1) in scramble, GCN5-KD, and NAA40-KD cells. $n = 3$ biological replicates/group. Statistical analysis was performed using two-way ANOVA with post hoc Tukey's multiple-comparisons test; ns non-significant. (**P**) Normalised levels (%label 6 h/%label 0 h) of the indicated aqueous metabolites in scramble, GCN5-KD, and NAA40-KD cells. $n = 3$ biological replicates/group. Statistical analysis was performed using a one-way ANOVA with post hoc Dunnett's multiple-comparisons test. All comparisons between KD and Scramble that are non-significant are not indicated. Data information: all data are presented as mean ± SEM. Source data are available online for this figure.

© The Author(s)

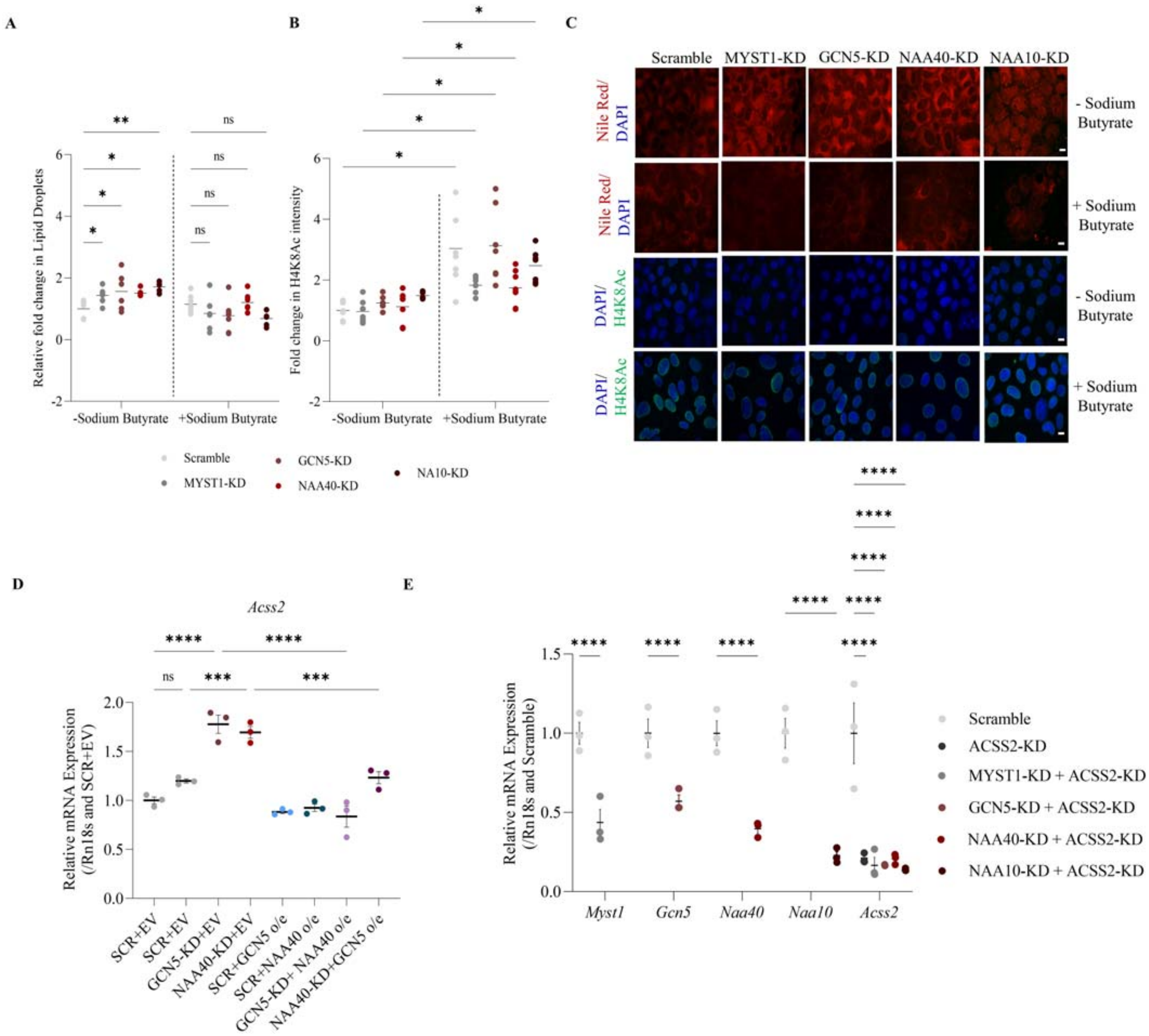

Figure EV4. Lipid synthesis upon acetyltransferase depletion is associated with ACSS2-driven HDAC-dependent acetate formation.

(**A**) Quantification of H4K8Ac using ImageJ MYST1-KD, GCN5-KD, NAA40-KD, and NAA10-KD cells with and without sodium butyrate, 48 h after siRNA treatment. $n = 6$ biological replicates/group. Statistical analysis was performed using two-way ANOVA with post hoc Tukey's multiple-comparisons test; *$P \leq 0.05$, **$P \leq 0.01$, ns non-significant. (**B**) Quantification of relative lipid droplets in scramble, MYST1-KD, GCN5-KD, NAA40-KD, and NAA10-KD cells with and without sodium butyrate, 48 h after siRNA treatment. $n = 6$ biological replicates/group. Statistical analysis was performed using two-way ANOVA with post hoc Tukey's multiple-comparisons test. Statistical analysis was performed using two-way ANOVA with post hoc Tukey's multiple-comparisons test; *$P \leq 0.05$. (**C**) Representative images of lipid droplets by Nile red staining (red), H4K8Ac (green) and nuclei by DAPI (blue) in scramble, MYST1-KD, GCN5-KD, NAA40-KD, and NAA10-KD cells with and without sodium butyrate, 48 h after siRNA treatment; Scale bar = 25 µm. $n = 6$ biological replicates/group. (**D**) RT-qPCR analysis of *Acss2* mRNA levels in the indicated treatment groups. $n = 3$ biological replicates/group. Statistical analysis was performed using a one-way ANOVA with post hoc Dunnett's multiple-comparisons test; ***$P \leq 0.001$, ****$P \leq 0.0001$, ns non-significant. (**E**) RT-qPCR analysis of *Myst-1*, *Gcn5*, *Naa40, Naa10* and *Acss2* mRNA levels in scramble, ACSS2-KD and the indicated double-KD cells after 48 h of siRNA treatment. $n = 3$ biological replicates/group. Statistical analysis was performed using two-way ANOVA with post hoc Tukey's multiple-comparisons test. ****$P \leq 0.0001$. Data information: all data are presented as mean ± SEM.

 © The Author(s)

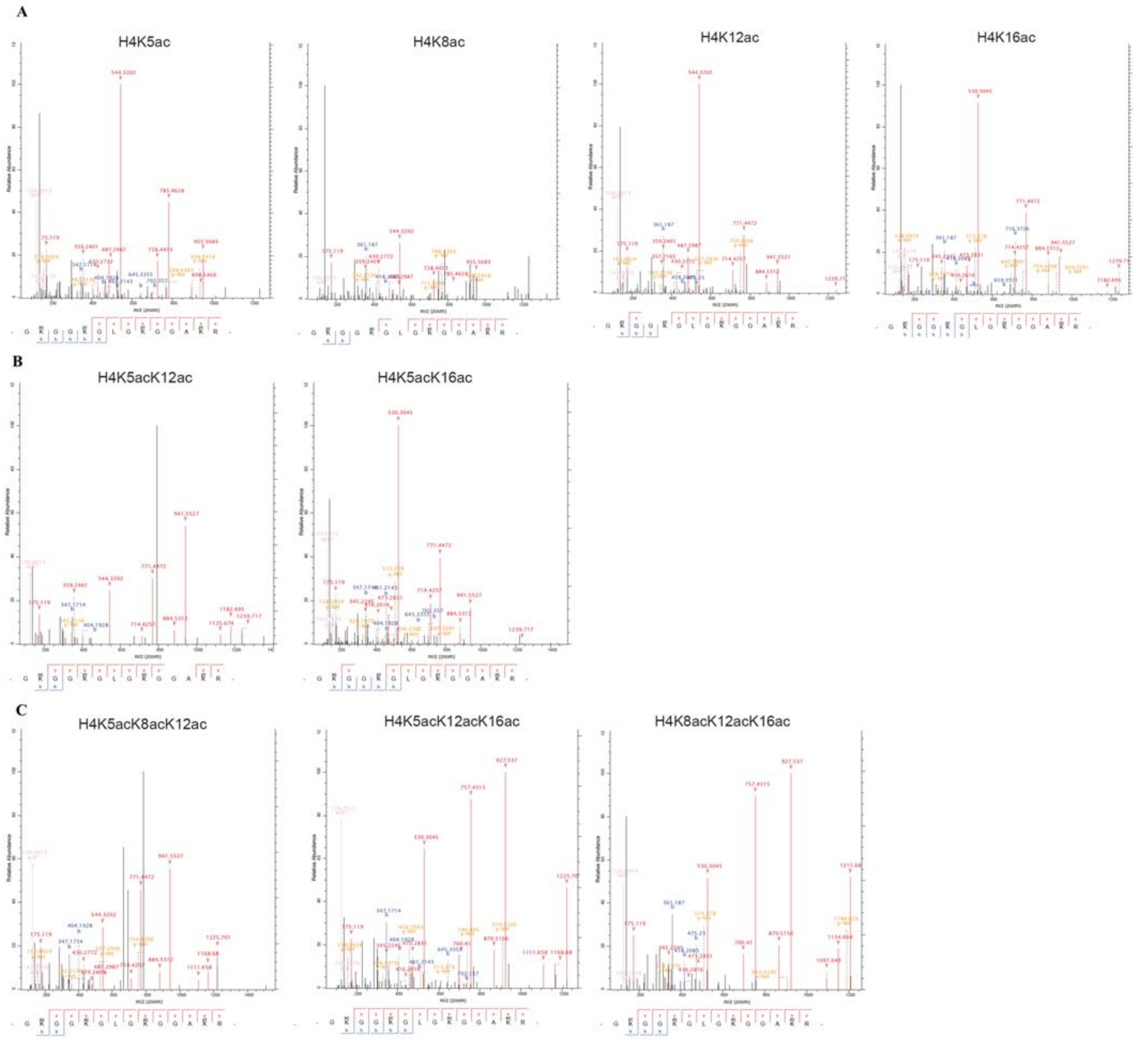

Figure EV5. MS-MS spectra for the differentially acetylated forms of the histone H4 tail.

(**A**) MS-MS spectra for differentially acetylated H4K5K8K12K16-1ac histone peptide. (**B**) MS-MS spectra for differentially acetylated H4K5K8K12K16-2ac histone peptide. (**C**) MS-MS spectra for differentially acetylated H4K5K8K12K16-3ac histone peptide.
